# Identification of putative biomarkers for prediabetes by metabolome analysis of rat models of type 2 diabetes

**DOI:** 10.1007/s11306-015-0784-9

**Published:** 2015-03-12

**Authors:** Norihide Yokoi, Masayuki Beppu, Eri Yoshida, Ritsuko Hoshikawa, Shihomi Hidaka, Toshiya Matsubara, Masami Shinohara, Yasuhiro Irino, Naoya Hatano, Susumu Seino

**Affiliations:** 1 Division of Molecular and Metabolic Medicine, Department of Physiology and Cell Biology, Kobe University Graduate School of Medicine, 7-5-1 Kusunoki-cho, Chuo-ku, Kobe, 650-0017 Japan; 2 Division of Cellular and Molecular Medicine, Department of Physiology and Cell Biology, Kobe University Graduate School of Medicine, Kobe, 650-0017 Japan; 3 Life Science Research Center, Technology Research Laboratory, Shimadzu Corporation, Kyoto, 619-0237 Japan; 4 Tokyo Animal and Diet Department, CLEA Japan, Inc., Meguro-ku, Tokyo, 153-8533 Japan; 5 The Integrated Center for Mass Spectrometry, Kobe University Graduate School of Medicine, Chuo-ku, Kobe, 650-0017 Japan; 6 Division of Evidenced-based Laboratory Medicine, Kobe University Graduate School of Medicine, Kobe, 650-0017 Japan

**Keywords:** Longitudinal study, Metabolomics, Plasma biomarkers, Rat models, Tryptophan, Type 2 diabetes

## Abstract

Biomarkers for the development of type 2 diabetes (T2D) are useful for prediction and intervention of the disease at earlier stages. In this study, we performed a longitudinal study of changes in metabolites using an animal model of T2D, the spontaneously diabetic Torii (SDT) rat. Fasting plasma samples of SDT and control Sprague-Dawley (SD) rats were collected from 6 to 24 weeks of age, and subjected to gas chromatography–mass spectrometry-based metabolome analysis. Fifty-nine hydrophilic metabolites were detected in plasma samples, including amino acids, carbohydrates, sugars and organic acids. At 12 weeks of age, just before the onset of diabetes in SDT rats, the amounts of nine of these metabolites (asparagine, glutamine, glycerol, kynurenine, mannose, n-alpha-acetyllysine, taurine, threonine, and tryptophan) in SDT rats were significantly different from those in SD rats. In particular, metabolites in the tryptophan metabolism pathway (tryptophan and kynurenine) were decreased in SDT rats at 12 weeks of age and later. The lower tryptophan and kynurenine levels in the prediabetic state and later were further confirmed by a replication study on SDT rats and by a longitudinal study on another animal model of T2D, the Otsuka Long-Evans Tokushima Fatty rat. Our data indicate that tryptophan and its metabolites are potential biomarkers for prediabetes and that tryptophan metabolism may be a potential target of intervention for treatment of the disease.

**Electronic supplementary material:**

The online version of this article (doi:10.1007/s11306-015-0784-9) contains supplementary material, which is available to authorized users.

## Introduction

Diabetes is a global health problem expected to afflict 592 million people by 2035 (International Diabetes Federation 2013). In the Western Pacific region including Japan, 138 million adults suffer from diabetes, the largest number of any region. Type 2 diabetes (T2D) comprises 90 % of diabetes worldwide (World Health Organization 1999). T2D is a metabolic disorder characterized by chronic hyperglycemia and associated with insulin resistance and/or impaired insulin secretion. While insulin resistance is thought to be a primary factor in the pathogenesis of T2D in Caucasian, Mexican–American, and Pima Indian populations (Haffner et al. 1996; Haffner et al. 1988), impaired insulin secretion has been reported to be a major factor in T2D in Japanese (Fukushima et al. 2004a, 2004b; Mitsui et al. 2006). Biomarkers for T2D, mainly in Caucasians, were reported including α-hydroxybutyrate (Gall et al. 2010), aromatic amino acids (Wang et al. 2011), triacylglycerols (Rhee et al. 2011), glycine and lysophosphatidylcholine (LPC) (Wang-Sattler et al. 2012), acyl-alkyl-phosphatidylcholines, diacylphosphatidylcholines, glycine, hexose, phenylalanine, and sphingomyelin (Floegel et al. 2013), and 2-aminoadipic acid (Wang et al. 2013). However, little is known of biomarkers for T2D in Asian populations including Japanese.

As metabolomics enables analysis of a global set of small molecule compounds (metabolites) in a biological sample that provides a unique measure of the physiological status of an organism, it is a powerful technology for identification of biomarkers. Several practical metabolomics platforms have been developed based on NMR, gas chromatography–mass spectrometry , liquid chromatography–mass spectrometry , and capillary electrophoresis–mass spectrometry (GC–MS, LC–MS, and CE–MS, respectively). Among them, GC–MS is a highly popular analytical system, and is increasingly becoming a common way to diagnose dozens of congenital metabolic diseases in earlier diagnosis (Kuhara 2001). Recently, a practical non-targeted GC–MS based metabolomics platform has been established (Tsugawa et al. 2011).

The spontaneously diabetic Torii (SDT) rat was established from normoglycemic Sprague-Dawley (SD) rats, and is a spontaneous animal model of T2D without obesity (Shinohara et al. 2000). Male SDT rats develop diabetes with 100 % incidence by 40 weeks of age. Before the onset of the disease, pathological changes such as inflammation and fibrosis occur in and around the pancreatic islets. These changes are accompanied by a decrease in the number of pancreatic β-cells, resulting in defects in insulin secretion (Masuyama et al. 2004; Sasase et al. 2013). Thus, SDT rats may well be a useful animal model for searching biomarkers of T2D with impaired insulin secretion.

In the present study, we performed GC–MS-based metabolome analysis of blood samples of SDT rats to identify potential biomarkers for T2D with impaired insulin secretion. We also performed a replication study on SDT rats and a longitudinal study on another animal model of T2D, Otsuka Long-Evans Tokushima Fatty (OLETF) rats.

## Materials and methods

### Animals

Male SDT rats [genetic background: Sprague-Dawley (SD) rat] were provided by CLEA Japan, Inc. (Tokyo, Japan). The control male SD rats were purchased from CLEA Japan, Inc. Male Otsuka Long-Evans Tokushima Fatty (OLETF) rats (genetic background: Long-Evans rat) and control male Long-Evans Tokushima Otsuka (LETO) rats (genetic background: Long-Evans rat) were provided by Hoshino Laboratory Animals, Inc. (Ibaraki, Japan). All animals were maintained under specific pathogen free conditions at 23 ± 2 °C and 55 ± 10 % relative humidity with a 12-h light–dark cycle, and were provided with water and a commercial diet CE-2 (CLEA Japan, Inc., Tokyo, Japan) at the Animal Facility of Kobe Biotechnology Research and Human Resource Development Center of Kobe University. All animal experiments were approved by the Committee on Animal Experimentation of Kobe University and carried out in accordance with the Guidelines for Animal Experimentation at Kobe University.

### Phenotyping and plasma collection

The SDT and SD rats were checked for body weight and blood glucose level by a portable glucose meter (ANTSENSE III, Bayer Medical, Tokyo, Japan) once a week from 6 to 24 weeks of age. Diabetes was defined as non-fasting blood glucose level equal to or higher than 300 mg/dl under ad libitum dietary conditions. At 6, 8, 12, 16, 20, and 24 weeks of age, whole blood samples were collected from the tarsal vein under pentobarbital anesthesia after an overnight (16 h) fast, and plasma was separated by centrifugation and stored at −80 °C for later metabolome analysis. In a different batch, SDT and SD rats were checked for body weight and blood glucose level from 6 to 24 weeks of age and fasting plasma samples were collected at 8, 12, and 20 weeks of age. In addition, OLETF and LETO rats were checked for body weight and blood glucose level from 6 to 36 weeks of age and fasting plasma samples were collected at 12, 20, 28, and 36 weeks of age.

### Intravenous glucose tolerance test (IVGTT)

At 12 weeks of age, IVGTT was performed on SDT and SD rats. After an overnight (16 h) fast, animals were anesthetized by intraperitoneal injection of pentobarbital sodium and subcutaneous injection of ketoprofen, and then two catheters filled with saline containing 10 units/ml of heparin were inserted into the jugular vein and femoral vein. A 50 % glucose solution was administered as a bolus injection at a dose of 1.0 g/kg body weight in basal state into jugular vein and blood samples (0.4 ml each) were taken from femoral vein at −6, −3, 1, 5, 12, 19, 30, 60, 90, and 120 min. Plasma volume was replaced by controlled normal saline infusion. Whole blood glucose level was immediately measured by the glucose oxidase method with an automated glucose analyzer (GA-1151, Arkray, Kyoto, Japan). Serum was separated by centrifugation and stored at −80 °C for later insulin measurement. Serum insulin level was measured by insulin ELISA kit (Shibayagi Co., Ltd., Gunma, Japan).

### Metabolome analysis

Low molecular weight hydrophilic metabolites were extracted using MeOH-CHCl_3_ method according to the previous reports (Nishiumi et al. 2012; Tsugawa et al. 2011). Briefly, 50 μl of serum were mixed with 250 μl of a solvent mixture (MeOH:H_2_O:CHCl_3_, 2.5:1:1, v/v/v) containing 20 μl of 0.25 mg/ml 2-isopropylmalic acid (Sigma-Aldrich, Tokyo, Japan) as an internal standard. The mixture was then shaken at 37 °C for 30 min and centrifuged at 16,000×*g* for 5 min at 4 °C. Then, 225 μl of supernatant was mixed with 200 μl of distilled water, and the solution centrifuged at 16,000×*g* for 5 min at 4 °C. The resultant supernatant (250 μl) containing hydrophilic primary metabolites was collected and lyophilized using a freeze dryer. For oximation, 40 μl of 20 mg/ml methoxyamine hydrochloride (Sigma-Aldrich, Tokyo, Japan) dissolved in pyridine was mixed with a lyophilized sample, and the mixture was then shaken at 30 °C for 90 min. For derivation, 20 μl of *N*-methyl-*N*-trimethylsilyl-trifluoroacetamide (MSTFA) (GL Science, Tokyo, Japan) was added, and the mixture was shaken at 37 °C for 30 min. The mixture was then centrifuged at 16,000×*g* for 5 min at 4 °C and the resultant supernatant was subjected to GC–MS analysis.

According to the previous reports (Nishiumi et al. 2012; Tsugawa et al. 2011), GC–MS analysis was performed using a GCMS-QP2010 Ultra (Shimadzu Co., Kyoto, Japan) with a fused silica capillary column (CP-SIL 8 CB low bleed/MS; 30 m × 0.25 mm inner diameter, film thickness: 0.25 μm; Agilent Co., Palo Alto, CA). The front inlet temperature was 230 °C. The flow rate of helium gas through the column was 39.0 cm/s. The column temperature was held at 80 °C for 2 min and then raised by 15 °C/min to 330 °C, and held there for 6 min. The transfer line and ion-source temperatures were 250 and 200 °C, respectively. Twenty scans per second were recorded over the mass range 85–500 *m*/*z* using the Advanced Scanning Speed Protocol (ASSP, Shimadzu Co.).

Data processing was performed according to the previous reports [Nishiumi et al. 2012; Tsugawa et al. 2011]. Briefly, raw data were exported in netCDF format and the peak detection and alignment were performed using the MetAlign software (Wageningen UR, The Netherlands). The resultant data were exported in CSV format, and then analyzed with in-house analytical software (AIoutput), which enables peak identification and semi-quantification using an in-house metabolite library. For semi-quantification, the peak height of a particular ion for each metabolite was normalized to the peak height of the specified ion of 2-isopropylmalic acid (internal standard). When multiple peaks were detected for a particular metabolite, which was mainly due to trimethylsilyl (TMS) derivatization and isomeric form, the peak with relatively higher intensity that therefore seemed to reflect the level of the metabolite was adopted for the subsequent analysis. The upper and lower limits of detection and the upper and lower dynamic range limits were reported previously (Tsugawa et al. 2011). The data on glucose measured by GC–MS has been provided, although it was overloaded (Supplementary Fig. 1).

### Statistical analysis

The data are expressed as mean ± SEM. Differences in body weights, blood glucose levels, and serum insulin levels were assessed using Welch’s *t* tests. For metabolomics data, principal component analysis (PCA) and orthogonal partial least square discriminant analysis (OPLS-DA) were performed on Pareto scaled data using SIMCA P+ 13.0 (Umetrics, Umeå, Sweden) and Welch’s *t* test was performed on raw data.

## Results

### Phenotypic characterization of SDT rats

**Fig. 1:**
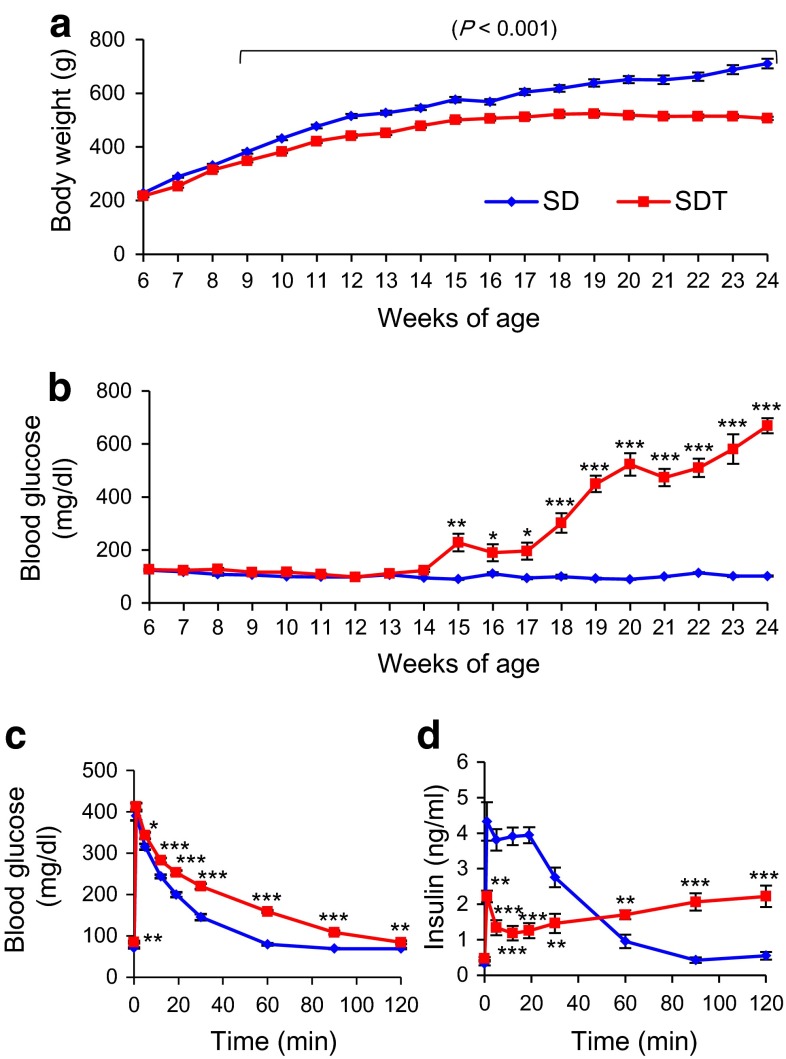
Phenotypic characterization of SDT and SD rats. Longitudinal changes in **a** body weight and **b** non-fasting blood glucose level from 6 to 24 weeks of age (n = 11 each). **c** blood glucose changes and **d** insulin response during IVGTT at 12 weeks of age (SD, n = 13; SDT, n = 10). The data are expressed as means ± SEM. Welch’s *t* test was used for evaluation of statistical significance. *p < 0.05, **p < 0.01, ***p < 0.001

We compared body weight and non-fasting blood glucose level of non-obese T2D model SDT rats and control SD rats from 6 to 24 weeks of age (Fig. 1a, b). Differences in body weight between SDT and SD rats were evident as early as 9 weeks of age (SDT 348.4 ± 5.8 vs. SD 381.7 ± 6.3 g, *p* = 0.0009), and increased gradually until 24 weeks of age (SDT 506.6 ± 6.2 vs. SD 710.6 ± 18.0 g, *p* < 0.0001). Differences in non-fasting blood glucose levels between SDT and SD rats were evident as early as 15 weeks of age (SDT 228.3 ± 33.4 vs. SD 90.2 ± 3.0 mg/dl, *p* = 0.002), and increased markedly until 24 weeks of age (SDT 668.8 ± 28.6 vs. SD 101.8 ± 2.4 mg/dl, *p* < 0.0001). SDT rats developed diabetes (non-fasting blood glucose levels ≥300 mg/dl) as early as 15 weeks of age and the cumulative incidence of diabetes reached 100 % by 20 weeks of age. In contrast, none of the SD rats developed diabetes.

To characterize the prediabetic state of SDT rats, we performed IVGTT at 12 weeks of age when there was yet no difference in non-fasting blood glucose levels between SDT and SD rats. SDT rats showed glucose intolerance accompanied with a defect in early phase insulin secretion (Fig. 1c, d). These results indicate that SDT rats exhibit a prediabetic state at 12 weeks of age, before the onset of overt diabetes.

### Longitudinal study of changes in metabolites between SDT and SD rats

**Fig. 2:**
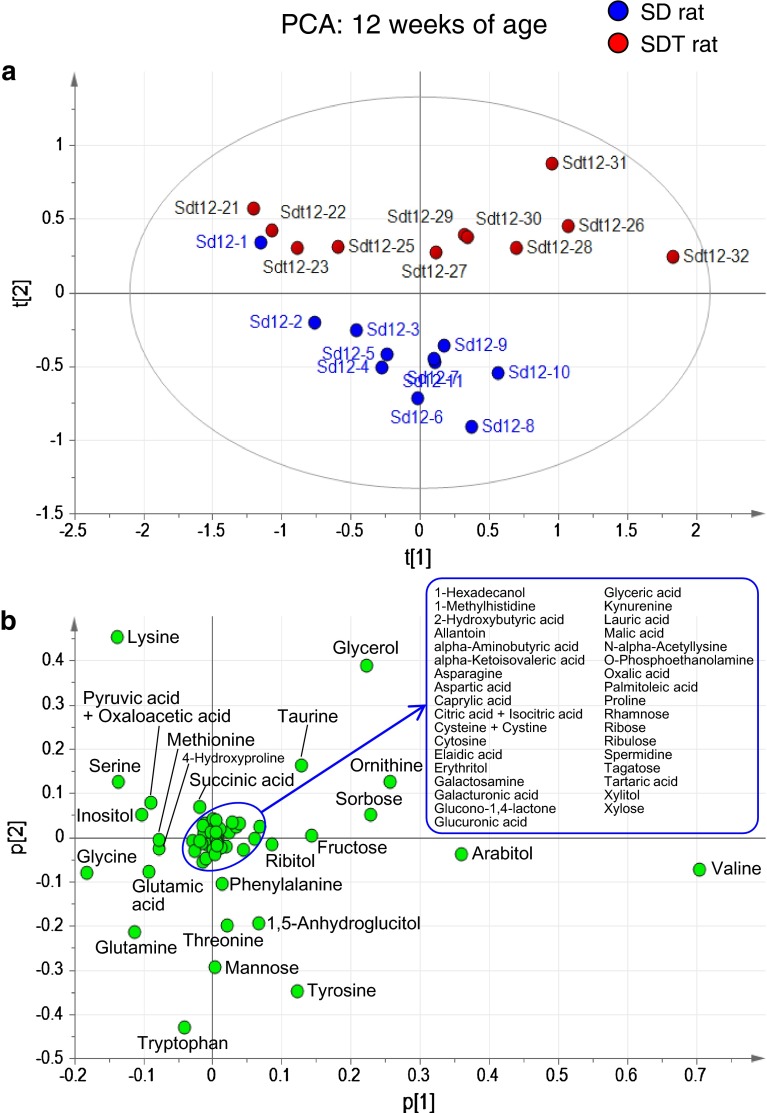

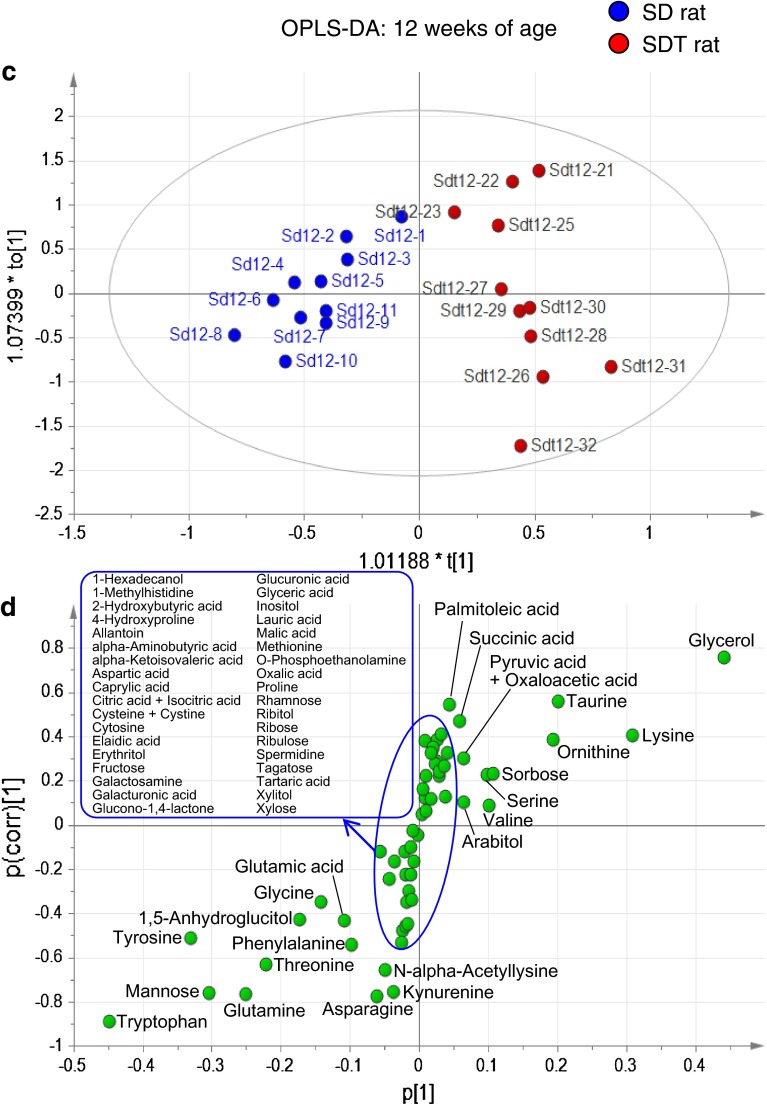
Metabolome analysis of plasma samples from SDT and SD rats at 12 weeks of age. **a** PCA score plot (PC1: 43.7 %; PC2: 17.5 %; R2: 61.3 %, Q2: 31.5 %), **b** PCA loading plot, **c** OPLS-DA score plot (R2: 87.6 %, Q2: 75.2 %), and **d** OPLS-DA S-plot of SD and SDT rats at 12 weeks of age

**Fig. 3:**
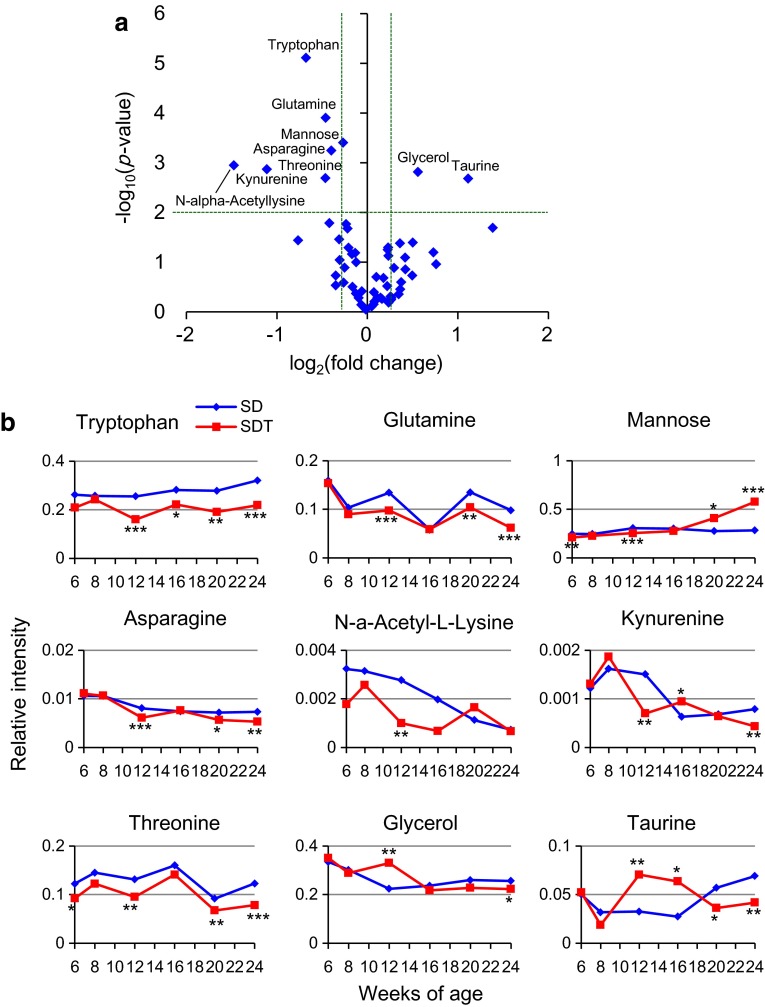
Comparison of content of each metabolite between SDT and SD rats. **a** Volcano plot of metabolome data on SDT and SD rats at 12 weeks of age. *x* axis shows log_2_(fold change calculated by the value of SDT divided by the value of SD) and *y* axis shows −log_10_(Welch’s *t* test *p*-value) between SDT and SD rats. Each *dot* represents one metabolite. *Dotted lines* shows 1.2 fold change and *p* = 0.01. Names of metabolite exhibiting significant differences (*p* < 0.01 and fold change >1.2) between SDT and SD rats are shown. **b** Longitudinal changes in metabolites showing significant differences between SDT and SD rats at 12 weeks of age (n = 8–11 each). Content of metabolite was expressed as intensity of each metabolite relative to that of internal standard. The data are expressed as means without SEM for clarity. Welch’s *t* test was used for evaluation of statistical significance. *p < 0.05, **p < 0.01, ***p < 0.001

**Fig. 4:**
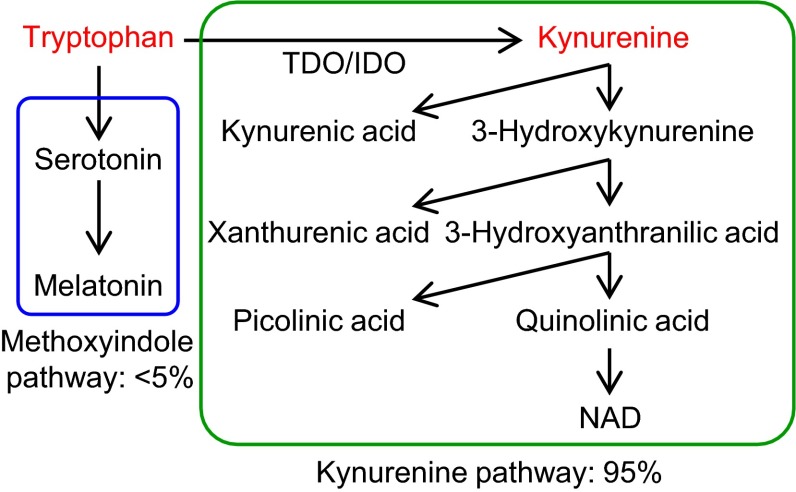
Tryptophan metabolism pathways. Tryptophan is metabolized through methoxyindole and kynurenine pathways: the latter is the major pathway of tryptophan metabolism

We performed GC–MS-based metabolome analysis on plasma samples of SDT and control SD rats. Fifty-nine hydrophilic metabolites were detected in plasma samples, including amino acids, carbohydrates, sugars and organic acids (Supplementary Table 1). PCA showed distinct metabolomic profiles by weeks of age: the age-dependent difference in profiles was more evident in SD rats than those in SDT rats (Supplementary Fig. 2). Difference in metabolomic profiles between SDT and SD rats was evident at 12 weeks of age, when SDT rats show a prediabetic state (Fig. 2a, b). OPLS-DA clearly indicated a higher amount of glycerol and a lower amount of tryptophan in SDT rats as compared to those in SD rats (Fig. 2c, d). The amounts of nine metabolites (asparagine, glutamine, glycerol, kynurenine, mannose, n-alpha-acetyllysine, taurine, threonine, and tryptophan) in SDT rats showed significant differences (*p* < 0.01; fold change >1.2) from those in SD rats (Fig. 3a). Longitudinal changes of these metabolites are shown in Fig. 3b. Among them, tryptophan was significantly decreased in SDT rats at 12 weeks of age and later, suggesting that tryptophan is a candidate biomarker for prediabetes. Tryptophan is metabolized through the methoxyindole pathway and also through the kynurenine pathway, the latter being the major pathway of tryptophan metabolism (Fig. 4). Kynurenine was significantly decreased in SDT rats at 12 and 24 weeks of age, but not at 16 or 20 weeks of age (Fig. 3b). These results indicate that tryptophan metabolism is changed at prediabetic state and later in SDT rats.

### Replication of the longitudinal study of SDT rats

**Fig. 5:**
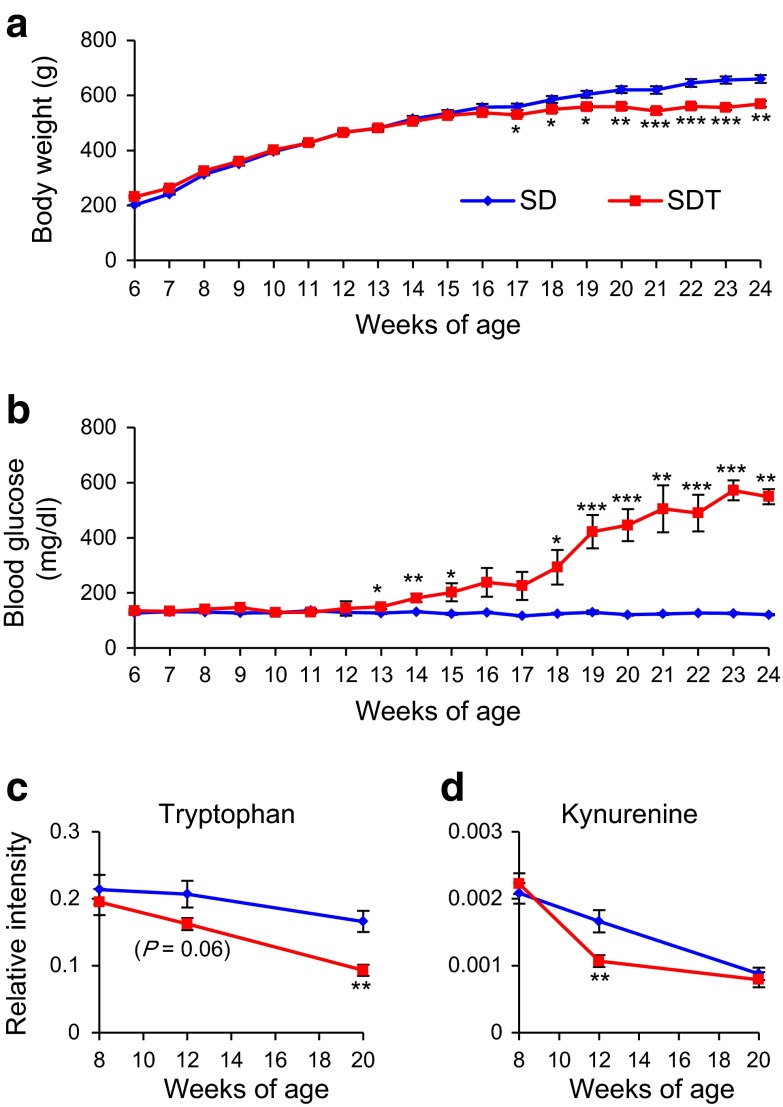
Replication study of SDT and SD rats. Longitudinal changes in **a** body weight and **b** non-fasting blood glucose level from 6 to 24 weeks of age (n = 10 each). Longitudinal changes in **c** tryptophan and **d** kynurenine levels (n = 10 each). The data are expressed as means ± SEM. Welch’s *t* test was used for evaluation of statistical significance. *p < 0.05, **p < 0.01, ***p < 0.001

To confirm these findings, we performed a replication study of SDT rats. We measured body weight and non-fasting blood glucose level of non-obese T2D model SDT rats and control SD rats from 6 to 24 weeks of age (Fig. 5a, b). Differences in body weight between SDT and SD rats were evident as early as 17 weeks of age, and increased gradually until 24 weeks of age (Fig. 5a). Differences in non-fasting blood glucose levels between SDT and SD rats were evident as early as 13 weeks of age, and increased markedly until 24 weeks of age (Fig. 5b). SDT rats developed diabetes as early as 14 weeks of age and the cumulative incidence of diabetes reached 100 % by 23 weeks of age. In contrast, none of the SD rats developed diabetes. Although chronological changes in body weight were not the same as those in the original study (Fig. 1a), the age of onset of diabetes was quite similar to that in the original study.

To compare the chronological changes in tryptophan and kynurenine levels in SDT and SD rats, we measured the contents of these metabolites in the replication study. Tryptophan showed a tendency to be decreased in SDT rats at 12 weeks of age and was significantly decreased at 20 weeks of age (Fig. 5c). Kynurenine was significantly decreased in SDT rats at 12 weeks of age, but not at 20 weeks of age (Fig. 5d). Chronological changes in tryptophan and kynurenine levels were confirmed by the replication study. These results indicate that tryptophan metabolism is already changed at prediabetic state in SDT rats.

### Longitudinal study of OLETF rats, another model of T2D

**Fig. 6:**
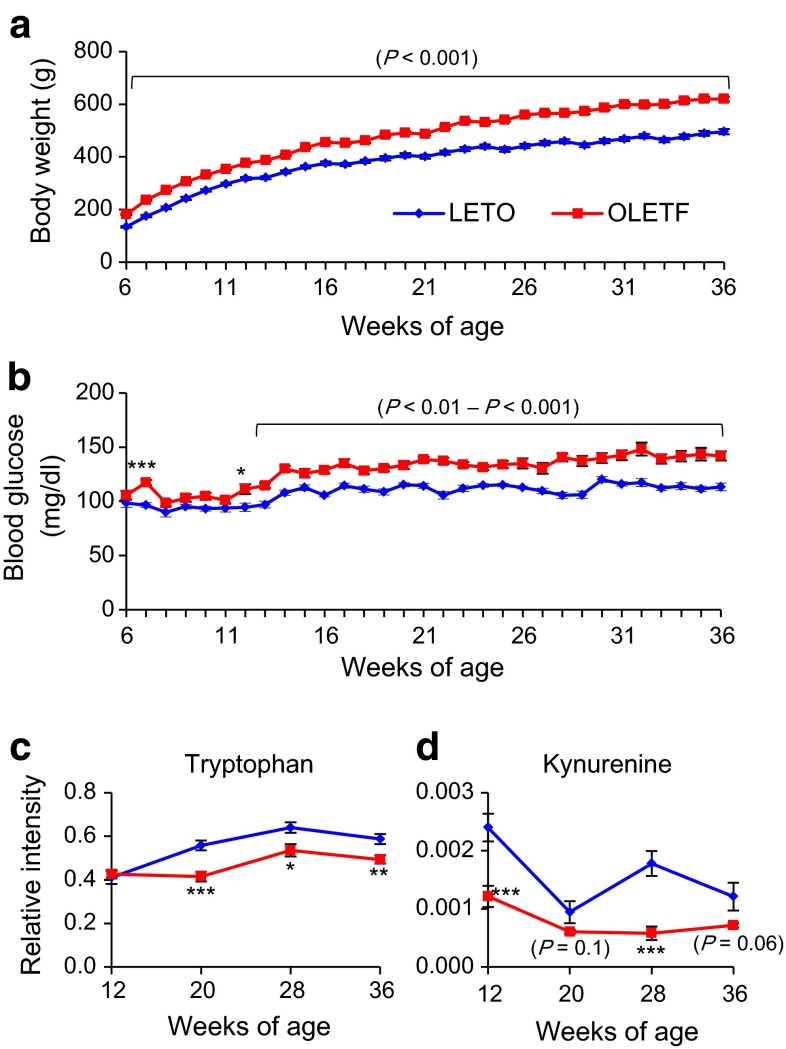
Phenotypic characterization and longitudinal changes of metabolites in OLETF and LETO rats. Longitudinal changes in **a** body weight and **b** non-fasting blood glucose level from 6 to 36 weeks of age (n = 12 each). Longitudinal changes in **c** tryptophan and **d** kynurenine levels (n = 12 each). The data are expressed as means ± SEM. Welch’s *t* test was used for evaluation of statistical significance. *p < 0.05, **p < 0.01, ***p < 0.001

To further examine involvement of tryptophan metabolism in T2D, we performed a longitudinal study on another animal model of T2D, the Otsuka Long-Evans Tokushima Fatty (OLETF) rat (Kawano et al. 1992). OLETF rats exhibit hyperphagia, obesity, insulin resistance, and impaired insulin secretion: most of the male rats are diagnosed with diabetes by oral glucose tolerance test (OGTT) at 25 weeks of age. Differences in body weight between OLETF and control LETO rats were evident as early as 6 weeks of age (OLETF 182.0 ± 2.8 vs. LETO 134.3 ± 2.6 g, *p* < 0.0001), and increased gradually until 36 weeks of age (OLETF 619.7 ± 9.3 vs. LETO 495.8 ± 10.0 g, *p* < 0.0001) (Fig. 6a). Differences in non-fasting blood glucose levels between OLETF and LETO rats were evident as early as 12 weeks of age (OLETF 111.3 ± 4.5 vs. LETO 94.6 ± 3.7 mg/dl, *p* = 0.01), and increased gradually until 36 weeks of age (OLETF 142.0 ± 4.2 vs. LETO 113.3 ± 3.6 mg/dl, *p* < 0.0001) (Fig. 6b). None of the OLETF rats showed severe hyperglycemia, indicating that the diabetic phenotype of OLETF rats is much milder than that of SDT rats.

We then compared the contents of tryptophan and kynurenine in OLETF and LETO rats. Tryptophan was significantly decreased in OLETF rats at 20 weeks of age and later (Fig. 6c). Kynurenine also was significantly decreased in OLETF rats at 12 and 28 weeks of age (Fig. 6d). Thus, chronological changes of tryptophan and kynurenine levels also are evident in another, different model of T2D. Together with the findings on SDT rats, these results suggest that tryptophan metabolism is already altered at prediabetic state in T2D.

## Discussion

By a longitudinal study of changes in metabolites in an animal model of T2D, SDT rats, we found that the amounts of tryptophan and kynurenine are decreased in the prediabetic state. These changes in tryptophan metabolism were confirmed by a replication study on SDT rats and a longitudinal study on another animal model of T2D, OLETF rats.

Tryptophan is an essential amino acid that can be metabolized through methoxyindole and kynurenine pathways (Fig. 4). In the former pathway, tryptophan serves as a precursor to serotonin and melatonin. Melatonin is considered to be an antioxidant. It was shown by studies of chemically-induced diabetic rats that melatonin increased antioxidant status (Sailaja Devi et al. 2000) and also suppressed hyperglycemia (Montilla et al. 1998). In addition, melatonin levels at night were found to be lower in T2D rats, and tryptophan administration raised the melatonin levels at night (Tormo et al. 2004). However, the role of the melatonin pathway in the pathogenesis and pathophysiology of SDT rats remains unknown.

The majority (~95 %) of tryptophan is metabolized by the kynurenine pathway, leading to the production of various metabolites including kynurenic acid and nicotinamide adenine dinucleotides (NAD). The rate-limiting enzymes of tryptophan to kynurenine conversion are tryptophan 2,3-dioxygenase (TDO) and indoleamine 2,3-dioxygenase (IDO) (Adam et al. 2005; Guillemin et al. 2001). TDO is mainly expressed in liver, kidney, and astrocyte in brain and the expression is induced by tryptophan and stress hormones. In contrast, IDO is expressed ubiquitously, and the expression is induced by pro-inflammatory cytokines (Oxenkrug 2010; Schrocksnadel et al. 2006). The kynurenine/tryptophan ratio is frequently used as an indicator of IDO activity (Brandacher et al. 2006). Lower circulating levels of tryptophan and a higher kynurenine/tryptophan ratio have been reported in morbidly obese subjects and overweight/obese subjects (Brandacher et al. 2006; Mangge et al. 2014). Upregulation of IDO activity due to chronic, low-grade systemic inflammation could be involved in these phenomena (Brandacher et al. 2007). Chronic, low-grade systemic inflammation is a potential pathway in the pathogenesis of T2D. Plasma levels of pro-inflammatory markers, such as C-reactive protein and IL-18, have been reported to be associated with increased risk of T2D (Freeman et al. 2002; Thorand et al. 2005). As the activity of IDO is regulated by pro-inflammatory cytokines, its substrate and product (tryptophan and kynurenine, respectively) could well be potential predictive biomarkers of T2D.

In the present study, a consistent decrease in the tryptophan level at prediabetic state and later was found in two different rat models of T2D. However, there was no increase in kynurenine/tryptophan ratio in these models (Supplementary Fig. 3), suggesting that mechanisms other than the upregulation of IDO activity might be involved. Changes in metabolites downstream of kynurenine and organs (or tissues) responsible for these changes should be examined in future study. Since tryptophan is obtained from diet, the effects of feeding with tryptophan-supplemented diet or tryptophan administration on physiological/pathophysiological states also should be investigated in these models. In addition, the pathophysiological relevance of tryptophan metabolism in T2D should be examined in prospective studies in humans.

Several biomarkers for prediabetes, mainly in Caucasians, have been reported to date. For example, branched chain amino acids (BCAA) were found to be biomarkers for diabetes, obesity, and insulin resistance (Newgard et al. 2009; Shah et al. 2012; Wang et al. 2011). However, tryptophan levels were not reported in the studies by Newgard et al. (2009) or Shah et al. (2012). A cohort study by Wang et al. (2011) suggested that the higher level of tryptophan is associated with a risk for future diabetes. We found in the present study that valine, a BCAA, was rather decreased in SDT rats especially in the diabetic state. The discrepancy between previous human studies and our present rat study could be due to the differences in the characteristic features of diabetes: the former is characterized by obesity and insulin resistance, the latter by impaired insulin secretion.

## Concluding remarks

Using a GC–MS-based metabolomics approach, we found that the content of the metabolites of tryptophan metabolism (tryptophan and kynurenine) were decreased at prediabetic state in two different animal models of T2D. Our data suggest that tryptophan metabolism may already be changed at prediabetic state in T2D, implicating tryptophan and its metabolites as biomarker candidates for prediabetes and suggesting that tryptophan metabolism could be a potential target of intervention for the disease. Longitudinal studies of changes in metabolites in spontaneous animal models are practically powerful for identification of biomarkers and for investigation of the pathophysiology of the disease.

## Electronic supplementary material

Below is the link to the electronic supplementary material.

Supplementary material 1 (PDF 517 kb)
