# Supplementary material for: Identification of putative biomarkers for prediabetes by metabolome analysis of rat models of type 2 diabetes
Source: Metabolomics. 2015 Mar 12;11(5):1277–86. doi: 10.1007/s11306-015-0784-9 (PMC4559098; doi:10.1007/s11306-015-0784-9)
Supplement: Supplementary file 1 — Supplementary material 1 (PDF 517 kb) [file 11306_2015_784_MOESM1_ESM.pdf]

Supplementary Materials

Supplementary Table 1
Contents of metabolites in SDT and SD rats.

Supplementary Fig. 1
Glucose levels measured by GC-MS.

Supplementary Fig. 2
PCA score plots and loading plots of metabolomics data on SDT and SD rats.

Supplementary Fig. 3
Kynurenine/tryptophan ratio.

Supplementary Table 1 Contents of metabolites in SDT and SD rats (n = 11 each)

No.	RT	Metabolite name	6 weeks of age					
			SD rat		SDT rat		t-test	Fold
			Mean	SE	Mean	SE	*p* -value	change
1	712.40	1,5-Anhydroglucitol	0.3524	0.0146	0.3423	0.0077	0.5519	0.97
2	759.85	1-Hexadecanol	0.0023	0.0002	0.0024	0.0003	0.7528	1.05
3	698.10	1-Methylhistidine	0.0045	0.0005	0.0047	0.0002	0.7072	1.04
4	331.20	2-Hydroxybutyric acid	0.0217	0.0032	0.0277	0.0034	0.2098	1.28
5	563.55	4-Hydroxyproline	0.1215	0.0083	0.1147	0.0069	0.5367	0.94
6	727.25	Allantoin	0.0724	0.0050	0.0764	0.0032	0.5075	1.06
7	362.20	alpha-Aminobutyric acid	0.0562	0.0052	0.0593	0.0021	0.5873	1.06
8	320.70	alpha-Ketoisovaleric acid	0.0025	0.0002	0.0025	0.0002	0.8932	0.99
9	651.00	Arabitol	0.1145	0.0255	0.1062	0.0232	0.8121	0.93
10	632.90	Asparagine	0.0106	0.0007	0.0111	0.0008	0.6451	1.05
11	559.60	Aspartic acid	0.0090	0.0007	0.0090	0.0012	0.9964	1.00
12	422.55	Caprylic acid	0.0026	0.0001	0.0032	0.0002	0.0351	1.22
13	696.70	Citric acid + Isocitric acid	0.6190	0.0157	0.7135	0.0086	<0.0001	1.15
14	881.45	Cysteine + Cystine	0.0175	0.0018	0.0113	0.0010	0.0104	0.65
15	565.65	Cytosine	0.0025	0.0004	0.0020	0.0003	0.3315	0.81
16	860.45	Elaidic acid	0.0010	0.0001	0.0015	0.0002	0.1172	1.47
17	550.25	Erythritol	0.0047	0.0004	0.0041	0.0001	0.1910	0.89
18	721.05	Fructose	0.0261	0.0018	0.0312	0.0027	0.1432	1.19
19	744.25	Galactosamine	0.0089	0.0009	0.0078	0.0014	0.4911	0.87
20	756.45	Galacturonic acid	0.0016	0.0002	0.0012	0.0001	0.0331	0.73
21	741.70	Glucono-1,4-lactone	0.0153	0.0014	0.0203	0.0005	0.0049	1.33
22	744.75	Glucuronic acid	0.0012	0.0001	0.0014	0.0001	0.0645	1.18
23	608.70	Glutamic acid	0.1005	0.0057	0.0972	0.0077	0.7375	0.97
24	680.05	Glutamine	0.1593	0.0047	0.1538	0.0067	0.5049	0.97
25	456.55	Glyceric acid	0.0016	0.0002	0.0021	0.0002	0.0919	1.33
26	423.20	Glycerol	0.3349	0.0221	0.3508	0.0245	0.6355	1.05
27	446.15	Glycine	0.4339	0.0147	0.3706	0.0148	0.0067	0.85
28	806.95	Inositol	0.1701	0.0091	0.2034	0.0095	0.0207	1.20
29	848.15	Kynurenine	0.0012	0.0001	0.0013	0.0001	0.5552	1.07
30	627.85	Lauric acid (Dodecanoic acid)	0.0038	0.0005	0.0045	0.0005	0.2977	1.21
31	741.90	Lysine	0.9380	0.0383	1.2895	0.0395	<0.0001	1.37
32	542.75	Malic acid	0.0027	0.0003	0.0057	0.0004	<0.0001	2.15
33	723.35	Mannose	0.2471	0.0071	0.2096	0.0069	0.0012	0.85
34	562.75	Methionine	0.0911	0.0049	0.0878	0.0031	0.5678	0.96
35	817.85	N-alpha-Acetyllysine	0.0032	0.0009	0.0018	0.0005	0.1678	0.55
36	683.45	O-Phosphoethanolamine	0.0037	0.0004	0.0042	0.0004	0.3246	1.15
37	698.25	Ornithine	0.2305	0.0177	0.2338	0.0078	0.8686	1.01
38	335.15	Oxalic acid	0.0047	0.0004	0.0065	0.0003	0.0030	1.38
39	786.65	Palmitoleic acid	0.0036	0.0006	0.0034	0.0006	0.8376	0.95
40	616.70	Phenylalanine	0.0922	0.0049	0.1079	0.0027	0.0134	1.17
41	442.55	Proline	0.0182	0.0012	0.0165	0.0009	0.2643	0.91
42	279.25	Pyruvic acid + Oxaloacetic acid	0.0318	0.0049	0.0410	0.0069	0.2912	1.29
43	656.35	Rhamnose	0.0012	0.0001	0.0013	0.0002	0.4951	1.13
44	653.50	Ribitol	0.0069	0.0015	0.0069	0.0014	0.9898	1.00
45	632.25	Ribose	0.0054	0.0014	0.0028	0.0003	0.0903	0.51
46	632.75	Ribulose	0.0024	0.0004	0.0029	0.0002	0.2436	1.24
47	474.05	Serine	0.5138	0.0299	0.4487	0.0221	0.0967	0.87
48	717.05	Sorbose	0.0794	0.0035	0.0934	0.0064	0.0773	1.18
49	868.55	Spermidine	0.0013	0.0002	0.0028	0.0005	0.0249	2.12
50	450.35	Succinic acid	0.0159	0.0010	0.0225	0.0006	<0.0001	1.42
51	715.10	Tagatose	0.0033	0.0006	0.0030	0.0006	0.7096	0.91
52	613.95	Tartaric acid	0.0009	0.0001	0.0015	0.0001	0.0027	1.67
53	635.15	Taurine	0.0501	0.0094	0.0522	0.0039	0.8412	1.04
54	438.90	Threonine	0.1223	0.0100	0.0922	0.0062	0.0211	0.75
55	858.10	Tryptophan	0.2618	0.0189	0.2088	0.0180	0.0563	0.80
56	750.40	Tyrosine	0.7670	0.0321	0.8425	0.0362	0.1362	1.10
57	390.15	Valine	1.4526	0.0904	1.2427	0.0614	0.0715	0.86
58	644.80	Xylitol	0.0095	0.0016	0.0075	0.0017	0.3857	0.78
59	621.70	Xylose	0.0016	0.0002	0.0019	0.0003	0.3473	1.20

Content of metabolite was expressed as intensity of each metabolite relative to that of internal standard (2-isopropylmalic acid).
Fold change was calculated for each metabolite by dividing the mean value of SDT rat by that of SD rat.

Supplementary Table 1 (Continued.) Contents of metabolites in SDT and SD rats (n = 11 each)

No.	RT	Metabolite name	8 weeks of age					
			SD rat		SDT rat		t-test	Fold
			Mean	SE	Mean	SE	*p* -value	change
1	712.40	1,5-Anhydroglucitol	0.3093	0.0154	0.2821	0.0085	0.1411	0.91
2	759.85	1-Hexadecanol	0.0023	0.0002	0.0023	0.0002	0.9189	1.01
3	698.10	1-Methylhistidine	0.0039	0.0002	0.0034	0.0004	0.3629	0.88
4	331.20	2-Hydroxybutyric acid	0.0173	0.0017	0.0166	0.0035	0.8604	0.96
5	563.55	4-Hydroxyproline	0.0817	0.0081	0.0834	0.0077	0.8762	1.02
6	727.25	Allantoin	0.0530	0.0033	0.0527	0.0036	0.9612	1.00
7	362.20	alpha-Aminobutyric acid	0.0413	0.0028	0.0384	0.0033	0.5165	0.93
8	320.70	alpha-Ketoisovaleric acid	0.0026	0.0001	0.0027	0.0001	0.6717	1.02
9	651.00	Arabitol	0.3659	0.0346	0.3540	0.0291	0.7950	0.97
10	632.90	Asparagine	0.0106	0.0011	0.0106	0.0007	0.9624	1.01
11	559.60	Aspartic acid	0.0057	0.0004	0.0074	0.0008	0.0778	1.31
12	422.55	Caprylic acid	0.0030	0.0001	0.0035	0.0002	0.0306	1.19
13	696.70	Citric acid + Isocitric acid	0.5662	0.0137	0.6447	0.0150	0.0010	1.14
14	881.45	Cysteine + Cystine	0.0143	0.0012	0.0121	0.0009	0.1584	0.85
15	565.65	Cytosine	0.0043	0.0002	0.0042	0.0003	0.7611	0.98
16	860.45	Elaidic acid	0.0012	0.0001	0.0014	0.0003	0.4325	1.21
17	550.25	Erythritol	0.0053	0.0003	0.0053	0.0002	0.8696	0.99
18	721.05	Fructose	0.0301	0.0019	0.0382	0.0027	0.0239	1.27
19	744.25	Galactosamine	0.0076	0.0009	0.0085	0.0012	0.5738	1.12
20	756.45	Galacturonic acid	0.0016	0.0001	0.0014	0.0001	0.1140	0.83
21	741.70	Glucono-1,4-lactone	0.0145	0.0024	0.0184	0.0024	0.2617	1.27
22	744.75	Glucuronic acid	0.0011	0.0001	0.0014	0.0002	0.1391	1.34
23	608.70	Glutamic acid	0.0820	0.0052	0.0908	0.0047	0.2276	1.11
24	680.05	Glutamine	0.1034	0.0073	0.0900	0.0066	0.1878	0.87
25	456.55	Glyceric acid	0.0015	0.0001	0.0017	0.0001	0.4065	1.10
26	423.20	Glycerol	0.3011	0.0238	0.2887	0.0161	0.6719	0.96
27	446.15	Glycine	0.3819	0.0142	0.4088	0.0563	0.6524	1.07
28	806.95	Inositol	0.1560	0.0137	0.1588	0.0086	0.8626	1.02
29	848.15	Kynurenine	0.0016	0.0002	0.0019	0.0001	0.2754	1.15
30	627.85	Lauric acid (Dodecanoic acid)	0.0048	0.0006	0.0052	0.0008	0.6515	1.10
31	741.90	Lysine	0.9166	0.0524	1.1914	0.0402	0.0005	1.30
32	542.75	Malic acid	0.0022	0.0004	0.0096	0.0018	0.0023	4.39
33	723.35	Mannose	0.2458	0.0078	0.2270	0.0054	0.0630	0.92
34	562.75	Methionine	0.0785	0.0056	0.0795	0.0050	0.8915	1.01
35	817.85	N-alpha-Acetyllysine	0.0031	0.0012	0.0026	0.0007	0.6897	0.82
36	683.45	O-Phosphoethanolamine	0.0027	0.0002	0.0034	0.0003	0.0890	1.23
37	698.25	Ornithine	0.1973	0.0103	0.1789	0.0121	0.2592	0.91
38	335.15	Oxalic acid	0.0058	0.0002	0.0067	0.0002	0.0009	1.16
39	786.65	Palmitoleic acid	0.0029	0.0004	0.0050	0.0014	0.1841	1.72
40	616.70	Phenylalanine	0.0825	0.0038	0.0945	0.0061	0.1154	1.15
41	442.55	Proline	0.0141	0.0016	0.0134	0.0014	0.7484	0.95
42	279.25	Pyruvic acid + Oxaloacetic acid	0.0292	0.0050	0.0743	0.0156	0.0172	2.54
43	656.35	Rhamnose	0.0018	0.0002	0.0022	0.0002	0.2536	1.20
44	653.50	Ribitol	0.0227	0.0022	0.0221	0.0021	0.8458	0.97
45	632.25	Ribose	0.0044	0.0003	0.0038	0.0008	0.4943	0.86
46	632.75	Ribulose	0.0025	0.0003	0.0022	0.0004	0.5369	0.88
47	474.05	Serine	0.3807	0.0282	0.3774	0.0307	0.9383	0.99
48	717.05	Sorbose	0.0901	0.0042	0.1098	0.0050	0.0068	1.22
49	868.55	Spermidine	0.0016	0.0003	0.0012	0.0001	0.2089	0.76
50	450.35	Succinic acid	0.0152	0.0013	0.0329	0.0042	0.0018	2.16
51	715.10	Tagatose	0.0029	0.0002	0.0028	0.0003	0.9117	0.99
52	613.95	Tartaric acid	0.0011	0.0001	0.0020	0.0002	0.0030	1.83
53	635.15	Taurine	0.0318	0.0067	0.0187	0.0056	0.1484	0.59
54	438.90	Threonine	0.1450	0.0100	0.1222	0.0088	0.1027	0.84
55	858.10	Tryptophan	0.2572	0.0176	0.2419	0.0134	0.4986	0.94
56	750.40	Tyrosine	0.7039	0.0229	0.6641	0.0379	0.3829	0.94
57	390.15	Valine	1.1764	0.0527	0.8045	0.1079	0.0076	0.68
58	644.80	Xylitol	0.0192	0.0014	0.0190	0.0015	0.9169	0.99
59	621.70	Xylose	0.0022	0.0003	0.0031	0.0005	0.1473	1.41

Content of metabolite was expressed as intensity of each metabolite relative to that of internal standard (2-isopropylmalic acid).
Fold change was calculated for each metabolite by dividing the mean value of SDT rat by that of SD rat.

Supplementary Table 1 (Continued.) Contents of metabolites in SDT and SD rats (n = 11 each)

No.	RT	Metabolite name	12 weeks of age					
			SD rat		SDT rat		t-test	Fold
			Mean	SE	Mean	SE	*p* -value	change
1	712.40	1,5-Anhydroglucitol	0.1524	0.0147	0.1235	0.0059	0.0902	0.81
2	759.85	1-Hexadecanol	0.0032	0.0002	0.0030	0.0003	0.5259	0.94
3	698.10	1-Methylhistidine	0.0029	0.0004	0.0041	0.0004	0.0403	1.42
4	331.20	2-Hydroxybutyric acid	0.0056	0.0005	0.0078	0.0015	0.1861	1.41
5	563.55	4-Hydroxyproline	0.0284	0.0020	0.0272	0.0028	0.7200	0.96
6	727.25	Allantoin	0.0640	0.0023	0.0588	0.0019	0.1004	0.92
7	362.20	alpha-Aminobutyric acid	0.0152	0.0013	0.0160	0.0011	0.6219	1.06
8	320.70	alpha-Ketoisovaleric acid	0.0017	0.0002	0.0014	0.0002	0.2598	0.83
9	651.00	Arabitol	0.1206	0.0233	0.1449	0.0346	0.5693	1.20
10	632.90	Asparagine	0.0080	0.0003	0.0061	0.0004	0.0006	0.76
11	559.60	Aspartic acid	0.0024	0.0002	0.0032	0.0004	0.0805	1.34
12	422.55	Caprylic acid	0.0036	0.0001	0.0042	0.0003	0.0561	1.17
13	696.70	Citric acid + Isocitric acid	0.4934	0.0175	0.4733	0.0143	0.3836	0.96
14	881.45	Cysteine + Cystine	0.0119	0.0011	0.0123	0.0010	0.7821	1.03
15	565.65	Cytosine	0.0014	0.0002	0.0011	0.0002	0.2940	0.79
16	860.45	Elaidic acid	0.0006	0.0001	0.0011	0.0003	0.1104	1.69
17	550.25	Erythritol	0.0041	0.0002	0.0035	0.0002	0.0172	0.85
18	721.05	Fructose	0.0472	0.0043	0.0529	0.0082	0.5494	1.12
19	744.25	Galactosamine	0.0068	0.0012	0.0081	0.0015	0.4888	1.20
20	756.45	Galacturonic acid	0.0012	0.0002	0.0007	0.00003	0.0365	0.59
21	741.70	Glucono-1,4-lactone	0.0236	0.0008	0.0249	0.0012	0.4054	1.05
22	744.75	Glucuronic acid	0.0009	0.0001	0.0008	0.0001	0.1284	0.84
23	608.70	Glutamic acid	0.0583	0.0039	0.0436	0.0041	0.0164	0.75
24	680.05	Glutamine	0.1341	0.0049	0.0975	0.0059	0.0001	0.73
25	456.55	Glyceric acid	0.0006	0.00004	0.0007	0.00002	0.0509	1.17
26	423.20	Glycerol	0.2240	0.0092	0.3302	0.0248	0.0015	1.47
27	446.15	Glycine	0.2767	0.0104	0.2460	0.0121	0.0687	0.89
28	806.95	Inositol	0.1073	0.0100	0.0983	0.0044	0.4262	0.92
29	848.15	Kynurenine	0.0015	0.0002	0.0007	0.00005	0.0014	0.46
30	627.85	Lauric acid (Dodecanoic acid)	0.0072	0.0003	0.0084	0.0006	0.0747	1.18
31	741.90	Lysine	1.0634	0.0467	1.1414	0.0350	0.1981	1.07
32	542.75	Malic acid	0.0014	0.0003	0.0016	0.0004	0.6296	1.18
33	723.35	Mannose	0.3057	0.0090	0.2542	0.0080	0.0004	0.83
34	562.75	Methionine	0.0573	0.0027	0.0566	0.0041	0.8905	0.99
35	817.85	N-alpha-Acetyllysine	0.0028	0.0004	0.0010	0.0002	0.0011	0.36
36	683.45	O-Phosphoethanolamine	0.0017	0.0001	0.0018	0.0002	0.5253	1.08
37	698.25	Ornithine	0.1812	0.0162	0.2330	0.0174	0.0419	1.29
38	335.15	Oxalic acid	0.0031	0.0006	0.0040	0.0006	0.2542	1.30
39	786.65	Palmitoleic acid	0.0010	0.0001	0.0025	0.0006	0.0205	2.61
40	616.70	Phenylalanine	0.0701	0.0026	0.0639	0.0019	0.0652	0.91
41	442.55	Proline	0.0076	0.0006	0.0068	0.0005	0.3133	0.89
42	279.25	Pyruvic acid + Oxaloacetic acid	0.0134	0.0019	0.0223	0.0040	0.0634	1.66
43	656.35	Rhamnose	0.0007	0.0001	0.0007	0.0001	0.5205	1.11
44	653.50	Ribitol	0.0067	0.0015	0.0079	0.0021	0.6491	1.18
45	632.25	Ribose	0.0018	0.0002	0.0022	0.0005	0.5256	1.20
46	632.75	Ribulose	0.0030	0.0001	0.0026	0.0001	0.0212	0.86
47	474.05	Serine	0.1850	0.0108	0.2097	0.0156	0.2089	1.13
48	717.05	Sorbose	0.1454	0.0101	0.1693	0.0200	0.3027	1.16
49	868.55	Spermidine	0.0015	0.0001	0.0012	0.0001	0.0349	0.81
50	450.35	Succinic acid	0.0107	0.0014	0.0132	0.0006	0.1309	1.23
51	715.10	Tagatose	0.0016	0.0001	0.0021	0.0003	0.1394	1.34
52	613.95	Tartaric acid	0.0007	0.0001	0.0009	0.0002	0.3519	1.29
53	635.15	Taurine	0.0326	0.0086	0.0705	0.0062	0.0021	2.16
54	438.90	Threonine	0.1311	0.0084	0.0951	0.0053	0.0020	0.73
55	858.10	Tryptophan	0.2554	0.0122	0.1598	0.0101	<0.0001	0.63
56	750.40	Tyrosine	0.6188	0.0349	0.5359	0.0178	0.0514	0.87
57	390.15	Valine	1.0593	0.0648	1.1124	0.1218	0.7058	1.05
58	644.80	Xylitol	0.0061	0.0015	0.0078	0.0015	0.4415	1.27
59	621.70	Xylose	0.0017	0.0001	0.0013	0.0002	0.1862	0.79

Content of metabolite was expressed as intensity of each metabolite relative to that of internal standard (2-isopropylmalic acid).
Fold change was calculated for each metabolite by dividing the mean value of SDT rat by that of SD rat.

Supplementary Table 1 (Continued.) Contents of metabolites in SDT and SD rats (n = 11 each)

No.	RT	Metabolite name	16 weeks of age					
			SD rat		SDT rat		t-test	Fold
			Mean	SE	Mean	SE	*p* -value	change
1	712.40	1,5-Anhydroglucitol	0.1254	0.0143	0.0325	0.0114	<0.0001	0.26
2	759.85	1-Hexadecanol	0.0031	0.0002	0.0027	0.0003	0.3419	0.88
3	698.10	1-Methylhistidine	0.0016	0.0003	0.0027	0.0005	0.0624	1.63
4	331.20	2-Hydroxybutyric acid	0.0032	0.0006	0.0035	0.0008	0.7700	1.10
5	563.55	4-Hydroxyproline	0.0141	0.0010	0.0189	0.0019	0.0400	1.34
6	727.25	Allantoin	0.0372	0.0013	0.0493	0.0033	0.0049	1.32
7	362.20	alpha-Aminobutyric acid	0.0101	0.0016	0.0115	0.0010	0.4697	1.14
8	320.70	alpha-Ketoisovaleric acid	0.0021	0.0001	0.0017	0.0002	0.0822	0.84
9	651.00	Arabitol	0.3878	0.0278	0.3925	0.0218	0.8936	1.01
10	632.90	Asparagine	0.0074	0.0008	0.0076	0.0007	0.8682	1.02
11	559.60	Aspartic acid	0.0018	0.0002	0.0046	0.0022	0.2440	2.50
12	422.55	Caprylic acid	0.0029	0.0002	0.0026	0.0002	0.1394	0.88
13	696.70	Citric acid + Isocitric acid	0.4443	0.0186	0.5062	0.0311	0.1066	1.14
14	881.45	Cysteine + Cystine	0.0090	0.0027	0.0102	0.0012	0.6888	1.14
15	565.65	Cytosine	0.0028	0.0002	0.0033	0.0002	0.0846	1.20
16	860.45	Elaidic acid	0.0007	0.0001	0.0008	0.0002	0.6010	1.14
17	550.25	Erythritol	0.0042	0.0002	0.0045	0.0002	0.2686	1.08
18	721.05	Fructose	0.1189	0.0301	0.1174	0.0193	0.9667	0.99
19	744.25	Galactosamine	0.0107	0.0023	0.0123	0.0020	0.6129	1.15
20	756.45	Galacturonic acid	0.0011	0.0001	0.0010	0.0001	0.3467	0.88
21	741.70	Glucono-1,4-lactone	0.0145	0.0032	0.0220	0.0029	0.0999	1.52
22	744.75	Glucuronic acid	0.0007	0.0001	0.0008	0.0001	0.1840	1.27
23	608.70	Glutamic acid	0.0433	0.0045	0.0510	0.0050	0.2653	1.18
24	680.05	Glutamine	0.0580	0.0067	0.0590	0.0061	0.9153	1.02
25	456.55	Glyceric acid	0.0014	0.0003	0.0013	0.0002	0.8597	0.96
26	423.20	Glycerol	0.2365	0.0116	0.2176	0.0249	0.5019	0.92
27	446.15	Glycine	0.2526	0.0112	0.2823	0.0105	0.0669	1.12
28	806.95	Inositol	0.0975	0.0042	0.1206	0.0144	0.1501	1.24
29	848.15	Kynurenine	0.0006	0.00004	0.0009	0.0001	0.0469	1.50
30	627.85	Lauric acid (Dodecanoic acid)	0.0087	0.0004	0.0086	0.0004	0.9038	0.99
31	741.90	Lysine	0.7120	0.0867	1.0161	0.0611	0.0102	1.43
32	542.75	Malic acid	0.0021	0.0004	0.0033	0.0007	0.1881	1.55
33	723.35	Mannose	0.3006	0.0148	0.2755	0.0091	0.1663	0.92
34	562.75	Methionine	0.0439	0.0035	0.0498	0.0046	0.3153	1.14
35	817.85	N-alpha-Acetyllysine	0.0020	0.0007	0.0007	0.0001	0.1090	0.34
36	683.45	O-Phosphoethanolamine	0.0013	0.0001	0.0022	0.0003	0.0232	1.63
37	698.25	Ornithine	0.0958	0.0098	0.1701	0.0182	0.0026	1.78
38	335.15	Oxalic acid	0.0043	0.0004	0.0054	0.0003	0.0494	1.25
39	786.65	Palmitoleic acid	0.0013	0.0003	0.0023	0.0013	0.4626	1.78
40	616.70	Phenylalanine	0.0604	0.0033	0.0716	0.0059	0.1153	1.19
41	442.55	Proline	0.0060	0.0004	0.0065	0.0007	0.5802	1.07
42	279.25	Pyruvic acid + Oxaloacetic acid	0.0073	0.0010	0.0163	0.0029	0.0116	2.23
43	656.35	Rhamnose	0.0007	0.0001	0.0011	0.0001	0.0237	1.54
44	653.50	Ribitol	0.0265	0.0022	0.0266	0.0018	0.9786	1.00
45	632.25	Ribose	0.0030	0.0003	0.0030	0.0004	0.9141	0.98
46	632.75	Ribulose	0.0023	0.0003	0.0026	0.0002	0.3985	1.15
47	474.05	Serine	0.1313	0.0098	0.1930	0.0173	0.0069	1.47
48	717.05	Sorbose	0.2835	0.0600	0.3005	0.0383	0.8147	1.06
49	868.55	Spermidine	0.0012	0.0001	0.0011	0.0001	0.2233	0.90
50	450.35	Succinic acid	0.0150	0.0012	0.0273	0.0056	0.0541	1.82
51	715.10	Tagatose	0.0019	0.0001	0.0026	0.0004	0.1370	1.39
52	613.95	Tartaric acid	0.0011	0.0001	0.0010	0.0001	0.6531	0.93
53	635.15	Taurine	0.0273	0.0090	0.0635	0.0141	0.0450	2.33
54	438.90	Threonine	0.1600	0.0136	0.1411	0.0135	0.3360	0.88
55	858.10	Tryptophan	0.2816	0.0212	0.2212	0.0156	0.0335	0.79
56	750.40	Tyrosine	0.5426	0.0299	0.5089	0.0282	0.4219	0.94
57	390.15	Valine	0.6991	0.0307	0.7433	0.0592	0.5173	1.06
58	644.80	Xylitol	0.0154	0.0017	0.0172	0.0010	0.3825	1.12
59	621.70	Xylose	0.0026	0.0004	0.0019	0.0004	0.2645	0.74

Content of metabolite was expressed as intensity of each metabolite relative to that of internal standard (2-isopropylmalic acid).
Fold change was calculated for each metabolite by dividing the mean value of SDT rat by that of SD rat.

Supplementary Table 1 (Continued.) Contents of metabolites in SDT and SD rats (n = 11 each)

No.	RT	Metabolite name	20 weeks of age					
			SD rat		SDT rat		t-test	Fold
			Mean	SE	Mean	SE	*p* -value	change
1	712.40	1,5-Anhydroglucitol	0.0440	0.0072	0.0008	0.00003	0.0001	0.02
2	759.85	1-Hexadecanol	0.0039	0.0002	0.0035	0.0003	0.3222	0.90
3	698.10	1-Methylhistidine	0.0009	0.0002	0.0011	0.0003	0.5466	1.22
4	331.20	2-Hydroxybutyric acid	0.0031	0.0006	0.0033	0.0006	0.8485	1.05
5	563.55	4-Hydroxyproline	0.0152	0.0012	0.0208	0.0014	0.0065	1.37
6	727.25	Allantoin	0.0547	0.0014	0.0597	0.0036	0.2240	1.09
7	362.20	alpha-Aminobutyric acid	0.0108	0.0020	0.0082	0.0006	0.2268	0.76
8	320.70	alpha-Ketoisovaleric acid	0.0012	0.0002	0.0014	0.0002	0.3368	1.23
9	651.00	Arabitol	0.6791	0.0403	0.6671	0.0207	0.7938	0.98
10	632.90	Asparagine	0.0072	0.0005	0.0056	0.0003	0.0159	0.79
11	559.60	Aspartic acid	0.0017	0.0001	0.0021	0.0001	0.0481	1.23
12	422.55	Caprylic acid	0.0037	0.0002	0.0030	0.0002	0.0245	0.80
13	696.70	Citric acid + Isocitric acid	0.4278	0.0215	0.4248	0.0116	0.9050	0.99
14	881.45	Cysteine + Cystine	0.0072	0.0007	0.0089	0.0013	0.2612	1.23
15	565.65	Cytosine	0.0016	0.0002	0.0016	0.0004	0.8750	0.96
16	860.45	Elaidic acid	0.0007	0.00004	0.0011	0.0001	0.0098	1.65
17	550.25	Erythritol	0.0047	0.0002	0.0052	0.0001	0.0518	1.10
18	721.05	Fructose	0.1580	0.0246	0.2474	0.0531	0.1490	1.57
19	744.25	Galactosamine	0.0083	0.0016	0.0304	0.0049	0.0010	3.67
20	756.45	Galacturonic acid	0.0009	0.0001	0.0010	0.0001	0.6699	1.05
21	741.70	Glucono-1,4-lactone	0.0185	0.0030	0.0191	0.0051	0.9212	1.03
22	744.75	Glucuronic acid	0.0006	0.00004	0.0010	0.0002	0.0456	1.59
23	608.70	Glutamic acid	0.0411	0.0032	0.0318	0.0027	0.0385	0.77
24	680.05	Glutamine	0.1349	0.0065	0.1038	0.0054	0.0015	0.77
25	456.55	Glyceric acid	0.0010	0.0001	0.0014	0.0002	0.0837	1.44
26	423.20	Glycerol	0.2596	0.0110	0.2281	0.0151	0.1090	0.88
27	446.15	Glycine	0.2204	0.0100	0.2305	0.0079	0.4345	1.05
28	806.95	Inositol	0.0843	0.0052	0.0927	0.0069	0.3427	1.10
29	848.15	Kynurenine	0.0007	0.00003	0.0006	0.0001	0.6226	0.94
30	627.85	Lauric acid (Dodecanoic acid)	0.0068	0.0005	0.0071	0.0004	0.6131	1.05
31	741.90	Lysine	0.6031	0.0290	0.8646	0.0879	0.0151	1.43
32	542.75	Malic acid	0.0015	0.0003	0.0041	0.0011	0.0451	2.73
33	723.35	Mannose	0.2754	0.0110	0.4093	0.0491	0.0222	1.49
34	562.75	Methionine	0.0505	0.0018	0.0369	0.0029	0.0011	0.73
35	817.85	N-alpha-Acetyllysine	0.0011	0.0002	0.0016	0.0005	0.3421	1.46
36	683.45	O-Phosphoethanolamine	0.0011	0.0001	0.0012	0.0001	0.6886	1.05
37	698.25	Ornithine	0.0816	0.0092	0.0900	0.0101	0.5436	1.10
38	335.15	Oxalic acid	0.0020	0.0004	0.0026	0.0004	0.3336	1.28
39	786.65	Palmitoleic acid	0.0009	0.0001	0.0010	0.0002	0.6317	1.11
40	616.70	Phenylalanine	0.0642	0.0030	0.0480	0.0029	0.0009	0.75
41	442.55	Proline	0.0082	0.0004	0.0065	0.0005	0.0097	0.79
42	279.25	Pyruvic acid + Oxaloacetic acid	0.0034	0.0004	0.0075	0.0007	0.0001	2.19
43	656.35	Rhamnose	0.0009	0.0001	0.0010	0.0001	0.4145	1.11
44	653.50	Ribitol	0.0454	0.0031	0.0459	0.0018	0.8926	1.01
45	632.25	Ribose	0.0031	0.0005	0.0033	0.0005	0.7213	1.08
46	632.75	Ribulose	0.0026	0.0003	0.0017	0.0003	0.0164	0.63
47	474.05	Serine	0.1604	0.0080	0.1979	0.0192	0.0940	1.23
48	717.05	Sorbose	0.3964	0.0469	0.5417	0.0856	0.1566	1.37
49	868.55	Spermidine	0.0013	0.0001	0.0013	0.0001	0.5935	1.03
50	450.35	Succinic acid	0.0239	0.0039	0.0245	0.0055	0.9267	1.03
51	715.10	Tagatose	0.0023	0.0001	0.0044	0.0007	0.0127	1.92
52	613.95	Tartaric acid	0.0010	0.0001	0.0009	0.0001	0.5336	0.93
53	635.15	Taurine	0.0570	0.0022	0.0362	0.0068	0.0128	0.63
54	438.90	Threonine	0.0916	0.0055	0.0674	0.0045	0.0029	0.74
55	858.10	Tryptophan	0.2780	0.0215	0.1907	0.0108	0.0025	0.69
56	750.40	Tyrosine	0.4273	0.0230	0.5024	0.0350	0.0908	1.18
57	390.15	Valine	0.6797	0.0413	0.5515	0.0377	0.0329	0.81
58	644.80	Xylitol	0.0228	0.0012	0.0240	0.0009	0.4227	1.05
59	621.70	Xylose	0.0025	0.0003	0.0031	0.0005	0.3154	1.24

Content of metabolite was expressed as intensity of each metabolite relative to that of internal standard (2-isopropylmalic acid).
Fold change was calculated for each metabolite by dividing the mean value of SDT rat by that of SD rat.

Supplementary Table 1 (Continued.) Contents of metabolites in SDT and SD rats (n = 11 each)

No.	RT	Metabolite name	24 weeks of age					
			SD rat		SDT rat		t-test	Fold
			Mean	SE	Mean	SE	*p* -value	change
1	712.40	1,5-Anhydroglucitol	0.0373	0.0051	0.0022	0.0007	0.0000	0.06
2	759.85	1-Hexadecanol	0.0032	0.0002	0.0026	0.0005	0.2441	0.80
3	698.10	1-Methylhistidine	0.0018	0.0004	0.0004	0.00003	0.0076	0.23
4	331.20	2-Hydroxybutyric acid	0.0055	0.0009	0.0043	0.0008	0.3553	0.78
5	563.55	4-Hydroxyproline	0.0159	0.0013	0.0182	0.0009	0.1674	1.15
6	727.25	Allantoin	0.0510	0.0020	0.0517	0.0024	0.8317	1.01
7	362.20	alpha-Aminobutyric acid	0.0148	0.0036	0.0069	0.0003	0.0540	0.47
8	320.70	alpha-Ketoisovaleric acid	0.0020	0.0001	0.0012	0.0001	<0.0001	0.60
9	651.00	Arabitol	0.9218	0.0536	0.7398	0.0375	0.0130	0.80
10	632.90	Asparagine	0.0073	0.0005	0.0053	0.0004	0.0068	0.72
11	559.60	Aspartic acid	0.0031	0.0003	0.0023	0.0003	0.0741	0.74
12	422.55	Caprylic acid	0.0032	0.0002	0.0019	0.0001	<0.0001	0.60
13	696.70	Citric acid + Isocitric acid	0.4763	0.0236	0.3633	0.0172	0.0013	0.76
14	881.45	Cysteine + Cystine	0.0102	0.0011	0.0144	0.0017	0.0616	1.40
15	565.65	Cytosine	0.0037	0.0002	0.0019	0.0002	<0.0001	0.50
16	860.45	Elaidic acid	0.0007	0.0000	0.0017	0.0002	0.0051	2.44
17	550.25	Erythritol	0.0057	0.0003	0.0059	0.0001	0.4970	1.03
18	721.05	Fructose	0.1780	0.0289	0.7112	0.1271	0.0037	4.00
19	744.25	Galactosamine	0.0180	0.0030	0.0436	0.0092	0.0283	2.43
20	756.45	Galacturonic acid	0.0011	0.0001	0.0015	0.0002	0.1081	1.37
21	741.70	Glucono-1,4-lactone	0.0160	0.0039	0.0148	0.0055	0.8568	0.92
22	744.75	Glucuronic acid	0.0008	0.0001	0.0018	0.0002	0.0030	2.20
23	608.70	Glutamic acid	0.0607	0.0052	0.0174	0.0009	<0.0001	0.29
24	680.05	Glutamine	0.0979	0.0063	0.0618	0.0047	0.0003	0.63
25	456.55	Glyceric acid	0.0012	0.0002	0.0042	0.0006	0.0014	3.37
26	423.20	Glycerol	0.2563	0.0103	0.2226	0.0117	0.0466	0.87
27	446.15	Glycine	0.2421	0.0072	0.2282	0.0080	0.2161	0.94
28	806.95	Inositol	0.0875	0.0037	0.1281	0.0098	0.0038	1.46
29	848.15	Kynurenine	0.0008	0.0001	0.0004	0.00003	0.0051	0.55
30	627.85	Lauric acid (Dodecanoic acid)	0.0083	0.0004	0.0063	0.0002	0.0003	0.76
31	741.90	Lysine	0.5944	0.0547	0.5267	0.0751	0.4781	0.89
32	542.75	Malic acid	0.0020	0.0005	0.0074	0.0016	0.0100	3.73
33	723.35	Mannose	0.2833	0.0128	0.5769	0.0532	0.0007	2.04
34	562.75	Methionine	0.0543	0.0024	0.0398	0.0014	0.0001	0.73
35	817.85	N-alpha-Acetyllysine	0.0007	0.0001	0.0007	0.0001	0.6793	0.91
36	683.45	O-Phosphoethanolamine	0.0017	0.0001	0.0010	0.0001	0.0007	0.57
37	698.25	Ornithine	0.1092	0.0167	0.0538	0.0081	0.0098	0.49
38	335.15	Oxalic acid	0.0025	0.0006	0.0034	0.0004	0.2072	1.36
39	786.65	Palmitoleic acid	0.0011	0.0001	0.0012	0.0002	0.8201	1.06
40	616.70	Phenylalanine	0.0725	0.0034	0.0503	0.0028	0.0001	0.69
41	442.55	Proline	0.0085	0.0006	0.0069	0.0004	0.0483	0.81
42	279.25	Pyruvic acid + Oxaloacetic acid	0.0037	0.0005	0.0022	0.0003	0.0238	0.61
43	656.35	Rhamnose	0.0018	0.0001	0.0015	0.0002	0.0963	0.81
44	653.50	Ribitol	0.0676	0.0047	0.0561	0.0030	0.0546	0.83
45	632.25	Ribose	0.0059	0.0004	0.0063	0.0006	0.5970	1.07
46	632.75	Ribulose	0.0026	0.0002	0.0012	0.0003	0.0011	0.46
47	474.05	Serine	0.1746	0.0088	0.1944	0.0136	0.2438	1.11
48	717.05	Sorbose	0.4497	0.0547	1.2842	0.1962	0.0034	2.86
49	868.55	Spermidine	0.0011	0.0001	0.0012	0.0001	0.8086	1.03
50	450.35	Succinic acid	0.0363	0.0048	0.0478	0.0092	0.2930	1.32
51	715.10	Tagatose	0.0025	0.0001	0.0085	0.0016	0.0062	3.44
52	613.95	Tartaric acid	0.0010	0.0001	0.0011	0.0001	0.6962	1.06
53	635.15	Taurine	0.0692	0.0044	0.0418	0.0061	0.0029	0.60
54	438.90	Threonine	0.1227	0.0055	0.0782	0.0046	<0.0001	0.64
55	858.10	Tryptophan	0.3206	0.0183	0.2186	0.0097	0.0002	0.68
56	750.40	Tyrosine	0.4801	0.0286	0.6726	0.0350	0.0007	1.40
57	390.15	Valine	0.9288	0.0858	0.6206	0.0278	0.0051	0.67
58	644.80	Xylitol	0.0301	0.0018	0.0243	0.0010	0.0155	0.81
59	621.70	Xylose	0.0043	0.0005	0.0050	0.0004	0.2462	1.17

Content of metabolite was expressed as intensity of each metabolite relative to that of internal standard (2-isopropylmalic acid).
Fold change was calculated for each metabolite by dividing the mean value of SDT rat by that of SD rat.

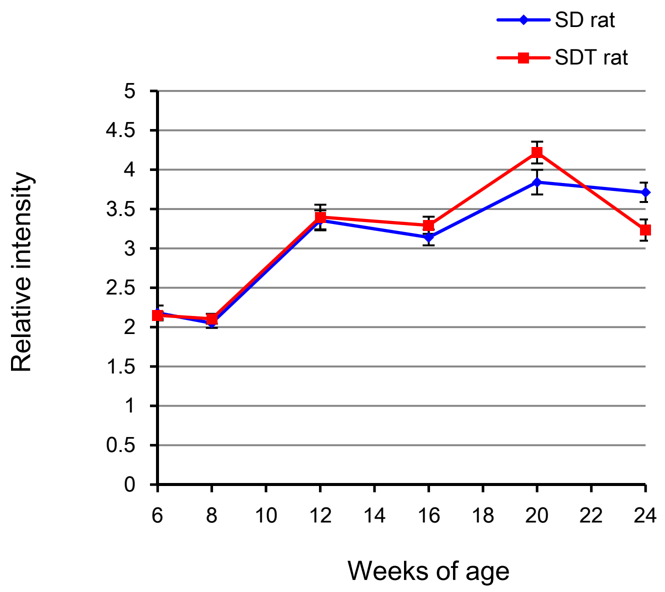

Supplementary Fig. 1

Glucose levels measured by GC-MS.

The data are expressed as means ± SEM.

Since the amount of glucose exceeded the upper limit of quantification, the difference in glucose levels between diabetic SDT and control SD rats could not be evaluated. A comparison between a GC-MS and a glucose meter with respect to sensitivity and repeatability was also provided:

GCMS-QP2010 Ultra

C.V.: < 5%

Sensitivity (See Tsugawa et al. 2011)

ANTSENSE III (portable glucose meter)

C.V.: < 5%

Lower limit: 10 mg/dl

Upper limit: 1,000 mg/dl

a

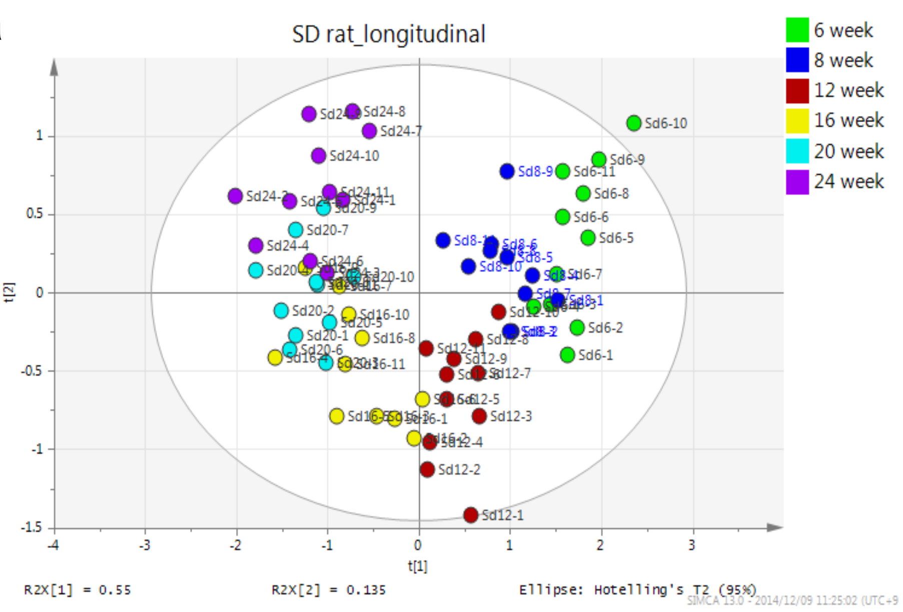

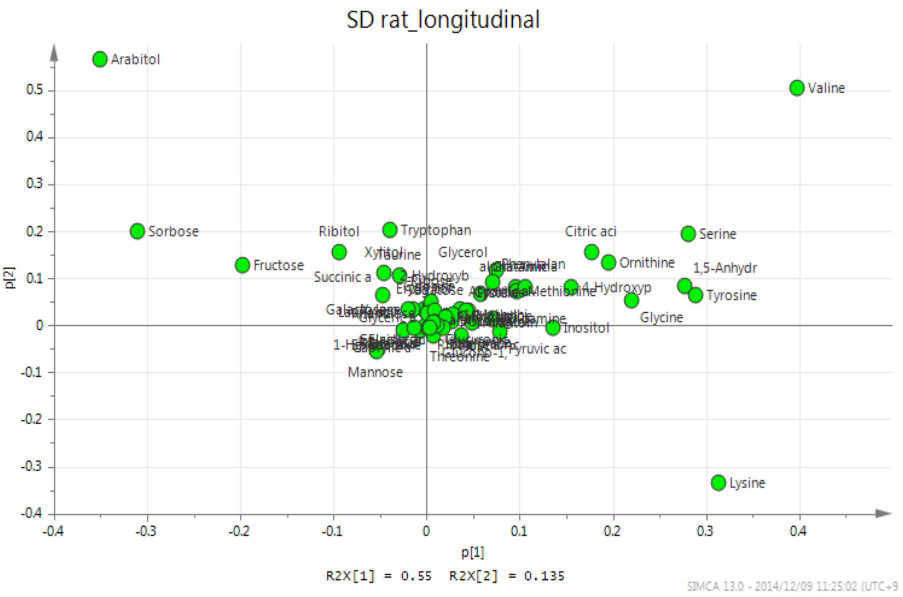

Supplementary Fig. 2

PCA score plots and loading plots of metabolomics data on SDT and SD rats.

PCA score plots and loading plots of **a** longitudinal samples from SD rats (PC1: 55.0%; PC2: 13.5%; R2: 68.5%, Q2: 51.8%), **b** longitudinal samples from SDT rats (PC1: 54.8%; PC2: 15.7%; R2: 70.5%, Q2: 56.4%), and samples from SDT and SD rats at **c** 6 weeks of age (PC1: 34.5%; PC2: 18.8%; R2: 53.3%, Q2: 8.8%), **d** 8 weeks of age (PC1: 30.7%; PC2: 22.1%; R2: 52.8%, Q2: 5.8%), **e** 16 weeks of age (PC1: 36.9%; PC2: 18.9%; R2: 55.8%, Q2: 2.3%), **f** 20 weeks of age (PC1: 33.2%; PC2: 21.2%; R2: 54.4%, Q2: 1.2%), and **g** 24 weeks of age (PC1: 60.5%; PC2: 15.1%; R2: 75.7%, Q2: 53.6%).

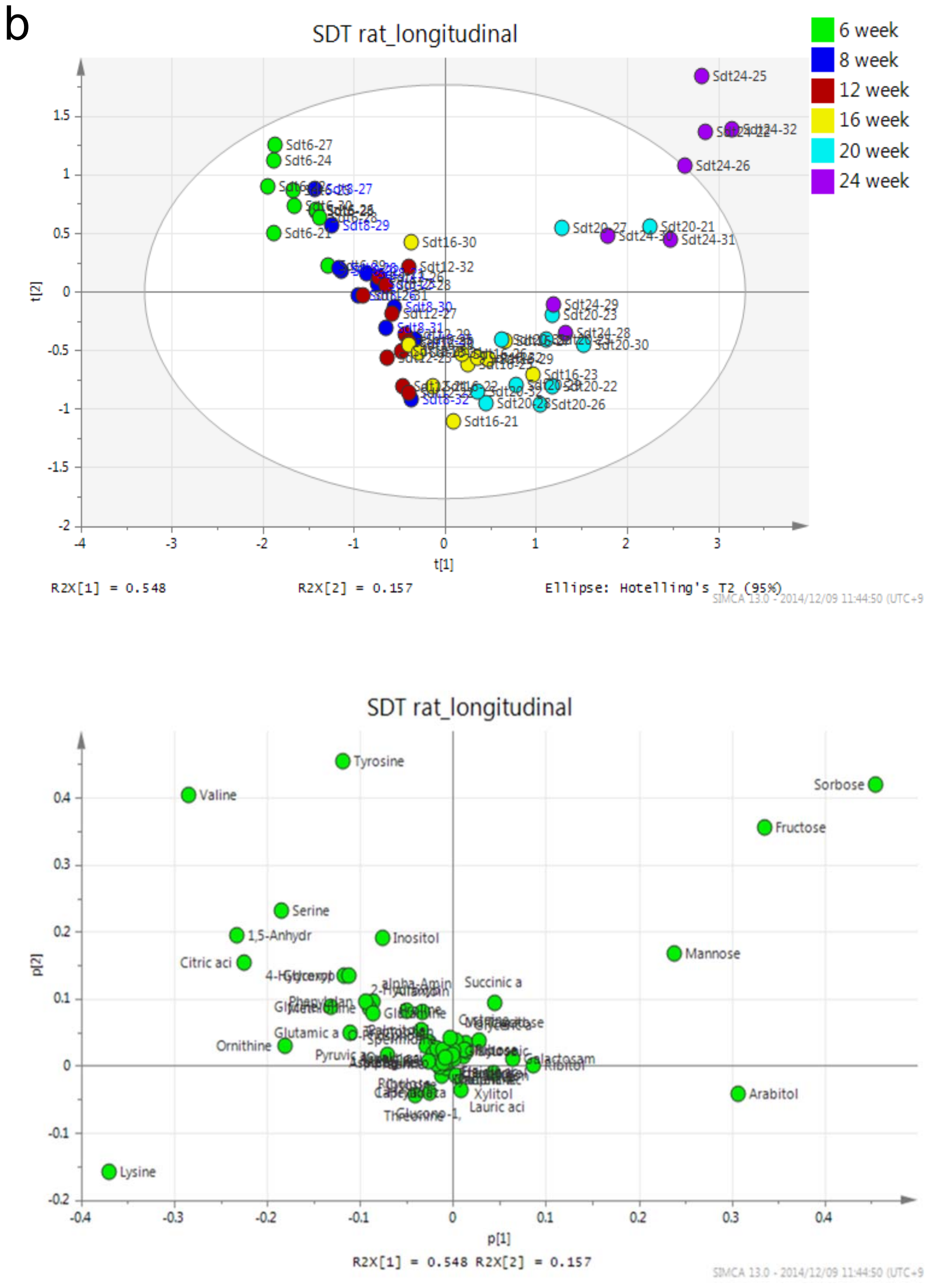

Supplementary Fig. 2 (Continued.)

C

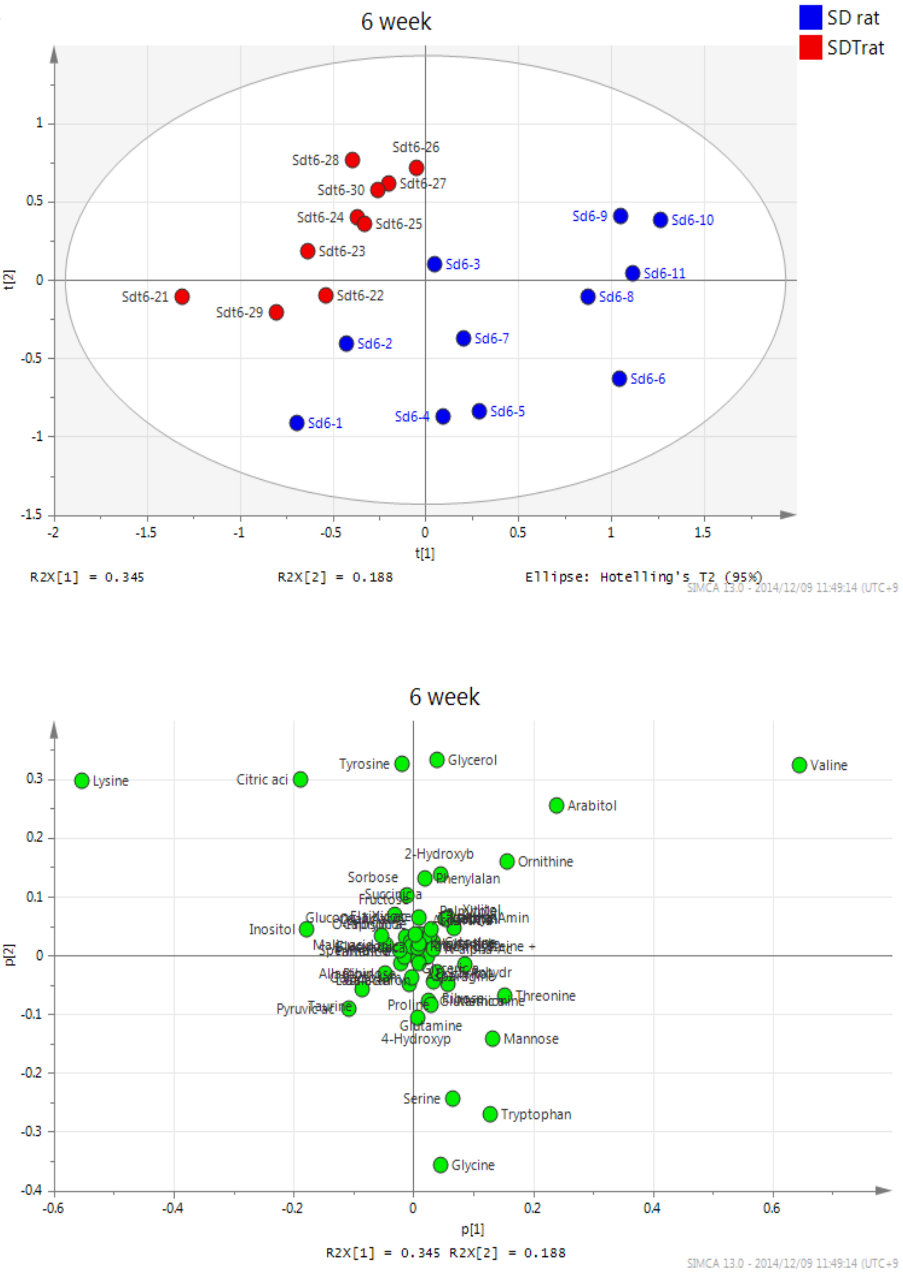

Supplementary Fig. 2 (Continued.)

d

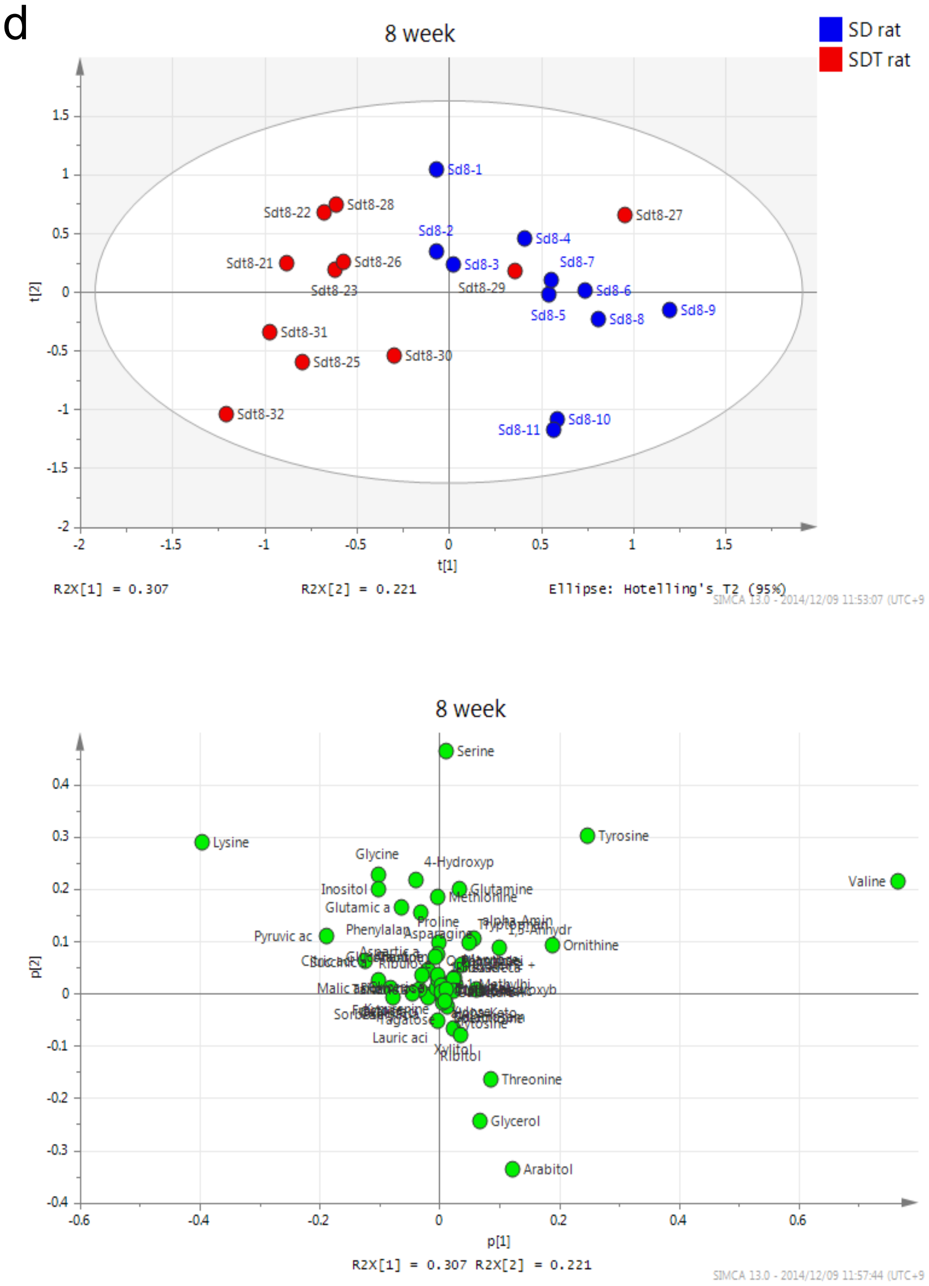

Supplementary Fig. 2 (Continued.)

e

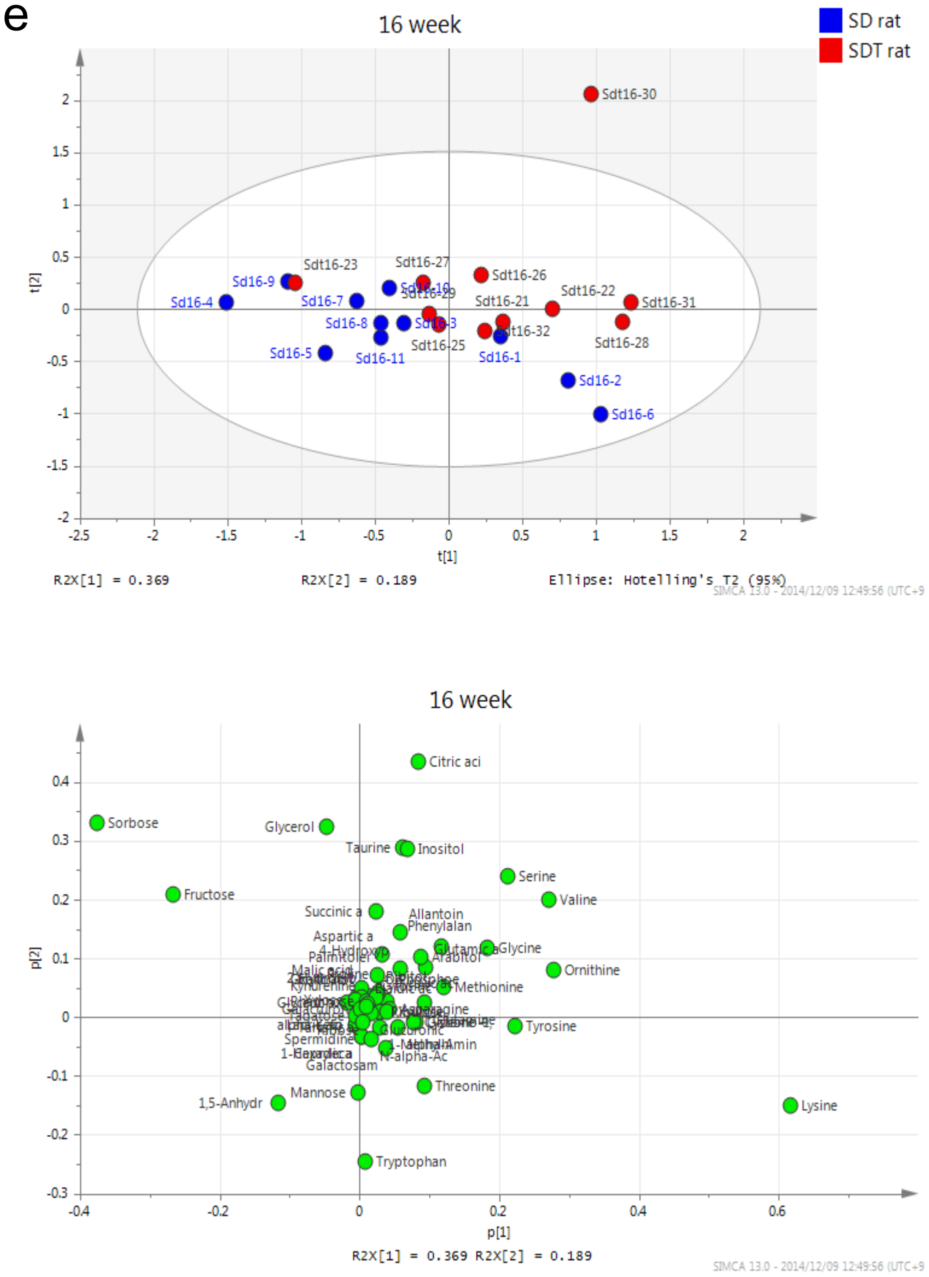

Supplementary Fig. 2 (Continued.)

f

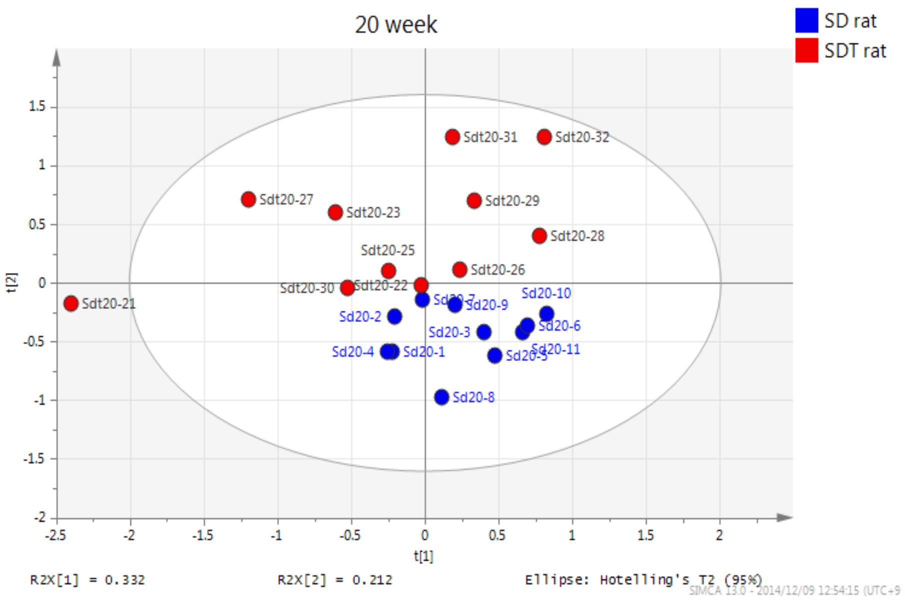

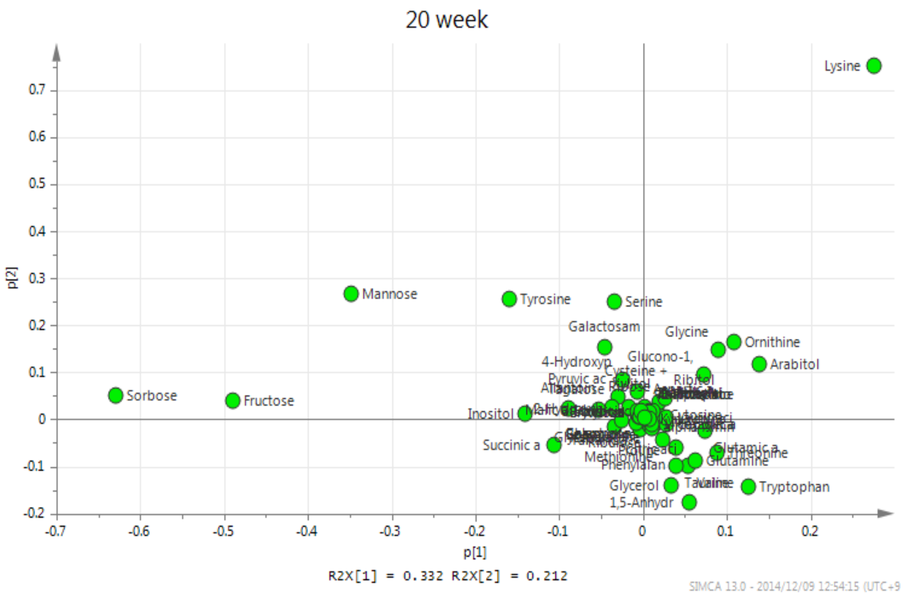

Supplementary Fig. 2 (Continued.)

g

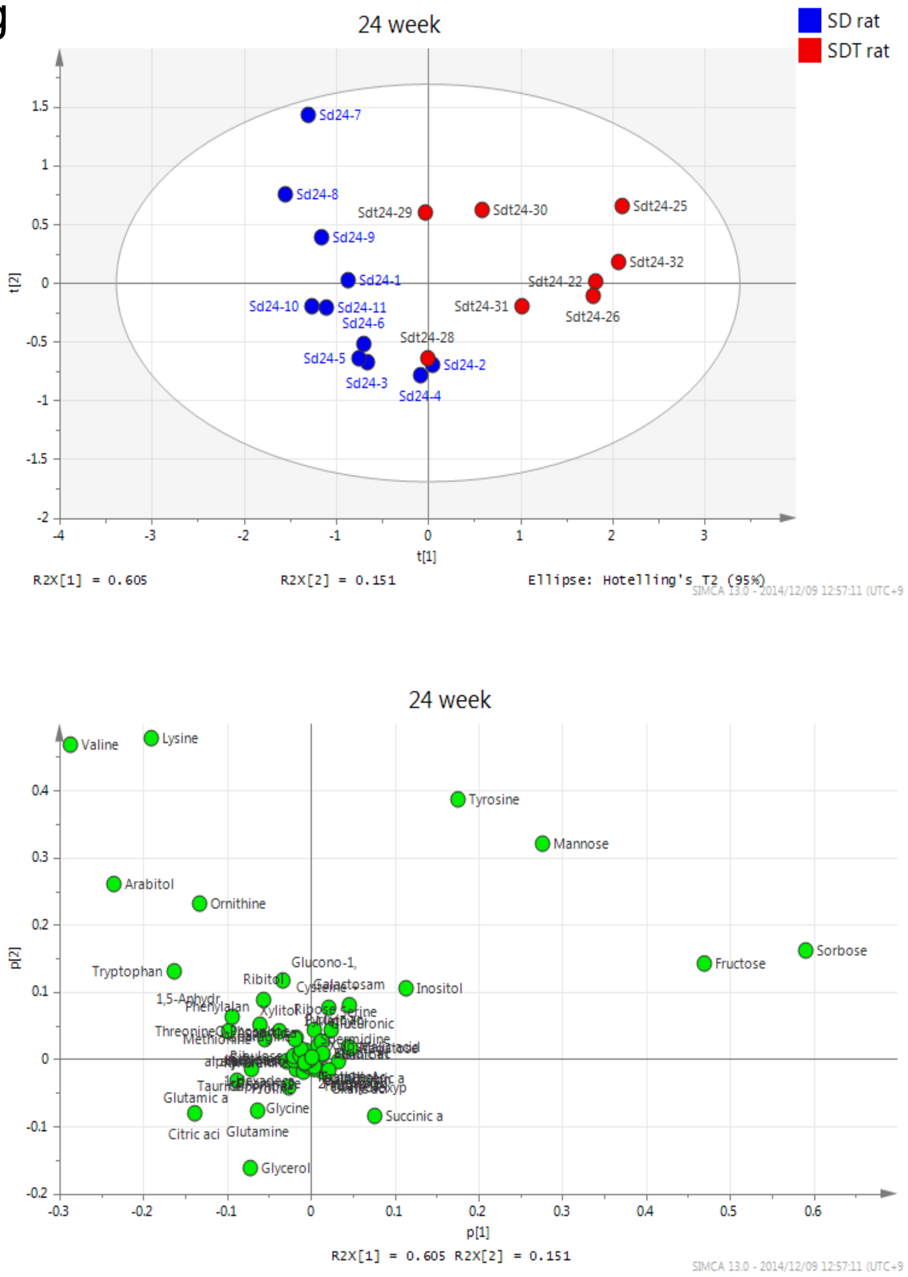

Supplementary Fig. 2 (Continued.)

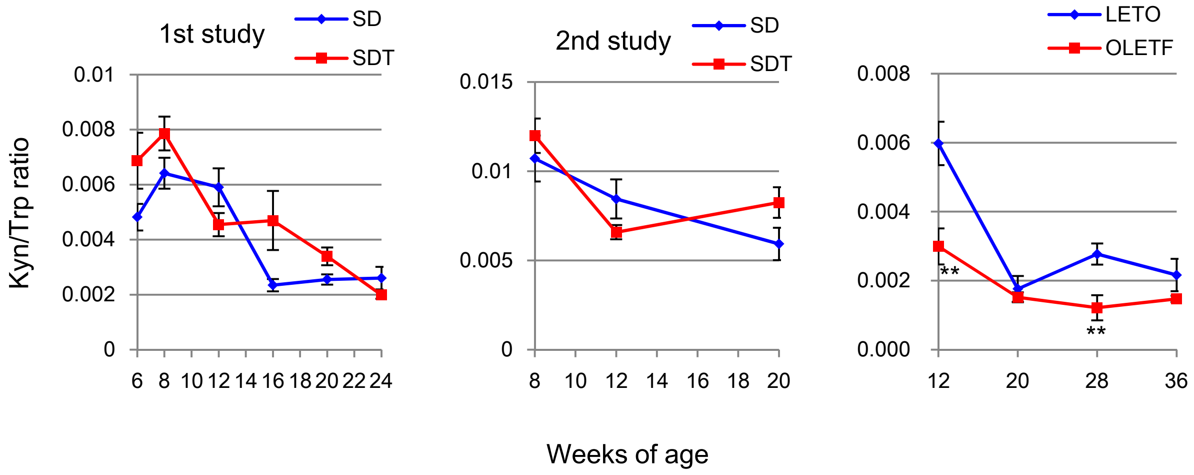

Supplementary Fig. 3

Kynurenine/tryptophan ratio.

The data are expressed as means ± SEM. Welch's t test was used for evaluation of statistical significance. ** $p < 0.01$
